# A mathematical model of the unfolded protein stress response reveals the decision mechanism for recovery, adaptation and apoptosis

**DOI:** 10.1186/1752-0509-7-16

**Published:** 2013-02-21

**Authors:** Kamil Erguler, Myrtani Pieri, Constantinos Deltas

**Affiliations:** 1 Molecular Medicine Research Center and Laboratory of Molecular and Medical Genetics, Department of Biological Sciences, University of Cyprus, Kallipoleos 75, 1678 Nicosia, Cyprus

**Keywords:** Endoplasmic reticulum stress, Unfolded protein response, Mathematical modelling, Translation attenuation, Chaperones, Bifurcation, Sensitivity, Oscillation

## Abstract

**Background:**

The unfolded protein response (UPR) is a major signalling cascade acting in the quality control of protein folding in the endoplasmic reticulum (ER). The cascade is known to play an accessory role in a range of genetic and environmental disorders including neurodegenerative and cardiovascular diseases, diabetes and kidney diseases. The three major receptors of the ER stress involved with the UPR, i.e. IRE1 *α*, PERK and ATF6, signal through a complex web of pathways to convey an appropriate response. The emerging behaviour ranges from adaptive to maladaptive depending on the severity of unfolded protein accumulation in the ER; however, the decision mechanism for the switch and its timing have so far been poorly understood.

**Results:**

Here, we propose a mechanism by which the UPR outcome switches between survival and death. We compose a mathematical model integrating the three signalling branches, and perform a comprehensive bifurcation analysis to investigate possible responses to stimuli. The analysis reveals three distinct states of behaviour, low, high and intermediate activity, associated with stress adaptation, tolerance, and the initiation of apoptosis. The decision to adapt or destruct can, therefore, be understood as a dynamic process where the balance between the stress and the folding capacity of the ER plays a pivotal role in managing the delivery of the most appropriate response. The model demonstrates for the first time that the UPR is capable of generating oscillations in translation attenuation and the apoptotic signals, and this is supplemented with a Bayesian sensitivity analysis identifying a set of parameters controlling this behaviour.

**Conclusions:**

This work contributes largely to the understanding of one of the most ubiquitous signalling pathways involved in protein folding quality control in the metazoan ER. The insights gained have direct consequences on the management of many UPR-related diseases, revealing, in addition, an extended list of candidate disease modifiers. Demonstration of stress adaptation sheds light to how preconditioning might be beneficial in manifesting the UPR outcome to prevent untimely apoptosis, and paves the way to novel approaches for the treatment of many UPR-related conditions.

## Background

Defects in protein folding might lead to the accumulation of unfolded or misfolded proteins in the endoplasmic reticulum (ER) causing stress, and the activation of the unfolded protein response (UPR) signalling cascade. The UPR is known to play an accessory role in a range of genetic and environmental disorders. It is particularly prominent in secretory cells as a bottleneck for the quality control of efficient and accurate protein folding and processing [1].

Glucose deprivation, disruption of calcium homeostasis, hypoxia and aging are known to induce ER stress and the UPR [2,3]. The UPR is also known to be involved in a range of neurological disorders such as Alzheimer’s, Parkinson’s and prion-related diseases [4], also in many others including type II diabetes, atherosclerosis and heart failure, amyotrophic lateral sclerosis (ALS), glomerulonephritis and acute kidney injury [5,6].

It has been demonstrated in a number of cases that manipulating the UPR improves the disease phenotype [1,7]. A noteworthy example is the process called preconditioning in which certain ER stress inducers are administered in order to favour an adaptive response, which prevents the destructive consequences of untimely apoptosis [8].

In order to understand better the modulating role of the UPR on many glomerulopathies, and other diseases with which it is involved, it is necessary to acquire a better picture of the mechanism of the UPR and its interactions with cellular disease mechanisms. On mammalian ER membrane there exists three well-known sensors for unmitigated unfolded protein accumulation: IRE1 *α*, PERK and ATF6 [9,10]. Each of these receptors is connected with a unique downstream pathway processing the stress signal into an appropriate response. The emerging behaviour ranges from adaptive, *i.e.* aiding protein folding and removing unfolded proteins, to maladaptive, *e.g.* pro-apoptotic, depending on the degree and the duration of unfolded protein accumulation [11].

Although each UPR pathway has been widely studied, the decision mechanism for switching between adaptive and maladaptive responses is yet to be uncovered. The differential responses of the three UPR branches against various stress sources and cross-links with other signalling pathways are also under investigation.

Here, we propose a literature-based mathematical model as a novel hypothesis which explains how the decision could be made to generate an appropriate response under prolonged stress conditions of various strengths. For the first time to our knowledge, the adaptive response mechanisms of the three signalling pathways, their cross-talk, and the associated genetic and post-translational interactions are being integrated into a coherent mechanistic model. The analysis of the resulting *in silico* UPR model reveals the different behavioural states that the UPR might undergo with respect to the strength and duration of the ER stress. The model demonstrates stress tolerance, adaptation and initiation of pro-apoptotic response profiles, and also suggests, contrary to prior expectations, that the UPR might turn gene expression on and off repeatedly under certain conditions.

## Results and discussion

### The detailed mechanistic model of the UPR

**Figure 1:**
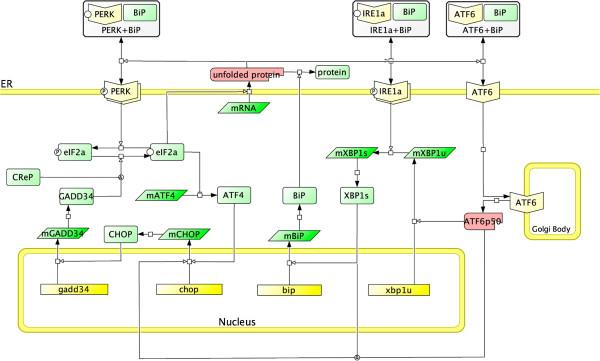
**The wiring diagram of the UPR model.** The complete UPR model comprises 27 species interconnected with 62 biochemical reaction channels in four compartments, ER, nucleus, Golgi body, and cytoplasm. The model utilises a total of 82 parameters. For sake of simplicity, some of the details and the apoptotic BAX/BAK/BH3 pathway have been omitted from the figure. Please refer to Additional file 1: Text 1 for the complete set of equations and parameters.

Here we construct a detailed ordinary differential equations (ODE) model of the UPR based on the recent literature [1,10,12,13]. The model comprises four main modules interconnected to each other. First of these is called the “receptor activation module”, which describes the dynamics of all the three membrane receptors, IRE1 *α*, PERK and ATF6, with regards to the unfolded protein (UFP) accumulation. The “translation attenuation module”, which is associated with PERK, describes the control of translation and the apoptotic signals. In addition, we describe two of the “adaptive response modules”, IRE1 *α* and ATF6 branches, which together control XBP1 dynamics and BiP synthesis. We present, in Figure 1, the simplified wiring diagram of the model outlining the compartments and components, and the reaction channels connecting them. The complete list of the model components, *i.e.* species, parameters (Additional file 1: Table S1) and reactions (Additional file 1: Table S2) can be seen in Additional file 1: Text 1. Throughout the text, we describe the main assumptions used in constructing the model, and in Additional file 1: Text 1, we present a summary to serve as a quick reference.

In this context, we focus on the cases of unmitigated ER stress, where the response mechanisms such as chaperone-assisted protein folding and ER-associated degradation (ERAD) are ineffective in reducing the amount or the rate of accumulation of UFP in the ER. Disconnecting the activation of the UPR from the response it generates emphasises the association between inputs and outputs, and therefore, permits an improved understanding of the decision mechanism. This way, we untangle the types and strengths of possible UPR outcomes — either of adaptive or maladaptive character — in response to a certain level of UFP.

The majority of the parameter values used in the model have not been measured experimentally. In addition, the data available from experimental studies on mammalian systems are not complete or sufficiently time-resolved making collective parameter inference a non-trivial task. We approach this problem with the aim of obtaining biologically plausible and testable predictions of qualitative behaviour. Rather than inferring a narrow range of parameter values, we aim to analyse a wide range of the parameter space. In accordance with this objective, we employ arbitrary units of time and concentration, *atu* and *acu* respectively, for the species and parameters of the model, and unless indicated otherwise we use them in the main and supplementary figures. Further studies designed to calibrate the model with experimental data for fine-tuned quantitative predictions will surely replace these with their canonical analogues.

In order to ease the analysis and circumvent the complexity, we investigate the system in four distinct modules. We perform bifurcation analyses for various parameters, investigate alternative models — testing the simplified versions where possible — and then, present the complete picture for which we verify the predictions with regards to experimental observations from literature. We present the modules in this section, and the analysis of the complete model in the following sections.

#### The receptor activation module

There are three main hypotheses for the activation of IRE1 in yeast: BiP binds to IRE1 monomers and prevents them from activation (no need for direct involvement of UFP), UFP binds directly to IRE1 and facilitates the activation (no need for direct involvement of BiP), or both BiP and UFP are involved in the activation [12]. A detailed mechanistic model developed by Pincus *et al.*[14] demonstrated that a mixture of both BiP and UFP regulation might come into effect in the activation of yeast IRE1 [15]. However, based on the differences in sequence between the luminal parts of yeast IRE1 and mammalian IRE1 *α*[10], the differences in their structure [16,17], and their differential abilities to prevent unfolded protein aggregation [18,19], we model the activation of the mammalian IRE1 *α* as dependent only on BiP. Compared to the mammalian IRE1 *α*, PERK has a closer evolutionary relationship to the yeast IRE1 [10]. Among the receptors, ATF6 is the least well-known with regards to the mechanism of its activation. Based on the accumulated evidence, we assume that BiP sequesters ATF6 while the unbound ATF6 is transported to the Golgi body without being oligomerised or phosphorylated [12].

**Figure 2:**
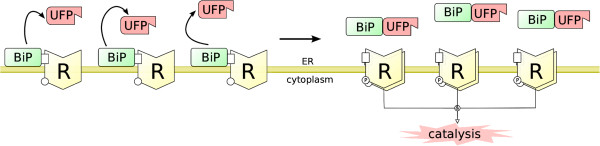
**The generic receptor activation module for the activation dynamics of IRE1 *α*, PERK and ATF6.** The receptor is shown with the symbol “R” with a cytoplasmic kinetic domain, and a luminal BiP-binding domain. Upon activation, the receptor oligomerises and autophosphorylates its cytoplasmic domain.

We aim to describe the receptor dynamics in a generic model applicable — with minor modifications — to all the three receptors (Figure 2). We assume that the control of activation is through competitive binding of BiP to the receptors and UFP, and also that the phosphorylated/active complex is capable of reversing to its inactive monomeric state without the need of an external phosphatase [20-22]. Since the protein-protein interactions are generally faster than the inflicted genetic regulatory steps, we assume these reactions take place at a faster pace — in a shorter time frame — than the rest of the system.

**Figure 3:**
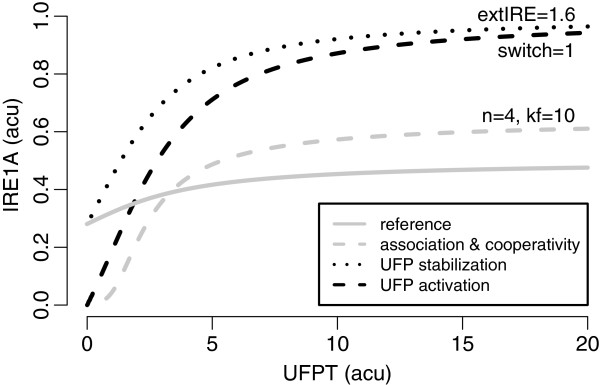
**Receptor activation dynamics under different parameterisation of the module.** The figure shows the total phosphorylated receptor (IRE1A) with respect to the total unfolded protein (UFPT). The grey solid curve corresponds to the reference parameter set given in Additional file 1: Text 1.1. The grey dashed curve describes the change from this when the rate of association and the stoichiometry of the activated complex are increased. The dark dotted and dashed curves refer to the effect of UFP stabilisation of the activated complex or its involvement in the direct activation, respectively.

Our standard model for activation is

$$\begin{array}{ll} \frac{d}{\text{dt}}{\left[\text{Receptor}\right]}_{\text{act}} & =v_{\text{act}}-v_{\text{deact}}\\ & =k_{f}\;{\left[\text{Receptor}\right]}^{n}-k_{r}\;{\left[\text{Receptor}\right]}_{\text{act}}, \end{array}$$

where *v*_act_ is the rate of oligomerisation and activation, *v*_deact_ is the rate of deactivation and dissociation, *k*_*f*_ and *k*_*r*_ are the rate constants for association and dissociation, respectively, and *n* is the stoichiometry of the activated complex. We hypothesise that UFP either directly assists in the oligomerisation and activation of the receptor, or it stabilises the activated receptor complex. We test the direct activation hypothesis with

$$v_{\text{act}}=k_{f}\;[\text{UFP}]\;{\left[\text{Receptor}\right]}^{n},$$

and the stabilisation hypothesis with

$$v_{\text{deact}}=\frac{k_{r}\;{\left[\text{Receptor}\right]}_{\text{act}}}{1+\text{extIRE}\;[\text{UFP}]},$$

where extIRE represents the strength of stabilisation. We observe that in both cases UFP elevates the resulting activation level; however, the basal activity is lower when UFP participates directly in the activation (the two black curves in Figure 3). In this case, increasing cooperativity delays the response, but results in a steeper threshold (Additional file 1: Figure S1(a)). If UFP stabilises the active complex, this will be sufficient to yield a rapid and large response (Additional file 1: Figure S1(b)).

It has been experimentally observed that the activation of IRE1 and PERK follow a steep response curve, and the process is highly cooperative [16,21,23-25]. Using a high association rate for the interactions of BiP, UFP and the membrane receptors, and also increasing the cooperativity of receptor activation, we demonstrate that it is possible to achieve rapid activation in response to UFP without its direct involvement (the grey dashed curve in Figure 3). In this case, the predicted activation dynamics includes a transient initial lag-phase as a result of cooperative binding, and the outcome is independent of direct UFP binding to the receptor. The effects of various parameter combinations can be seen in Additional file 1: Figures S2(a and b).

As a result, the model demonstrates a steep response curve with a brief lag-phase complying with experimental observations with the help of receptor association, *k*_*f*_, and cooperativity, *n*, parameters without strictly requiring the direct involvement of UFP. Since there is not enough data in the literature to justify, mechanistically, the direct involvement of UFP in receptor activation, we assume, in this context, the standard activation model without UFP involvement.

#### The IRE1 *α* branch

**Figure 4:**
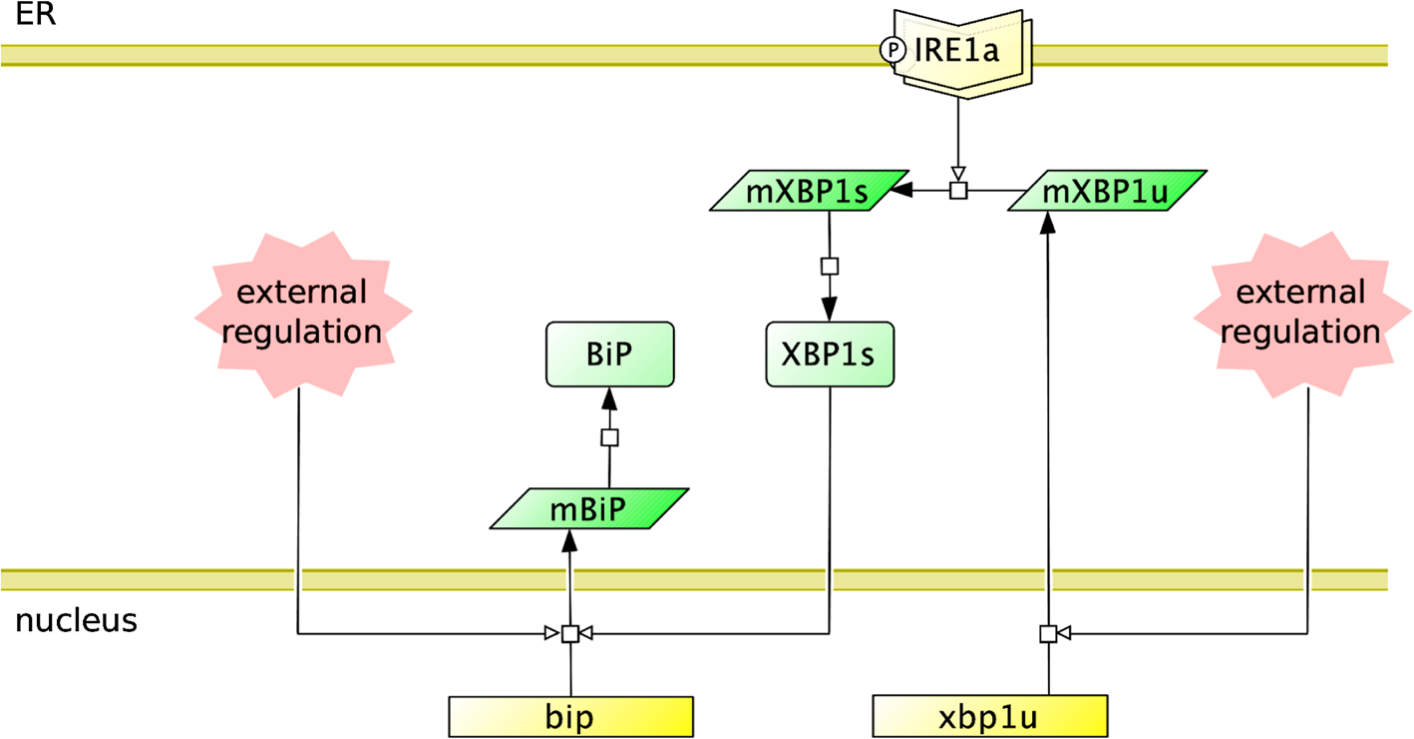
**The IRE1*α* branch of the adaptive response module.** The figure shows the activated IRE1 *α* cleaving the XBP1 mRNA (mXBP1) to yield the spliced XBP1 mRNA (mXBP1s), which translates into the XBP1 protein (XBP1s). There are two sites where the module interacts with external modules (with the ATF6 branch in the model) through regulation. These are drawn in the figure as the external regulators of BiP and XBP1 transcription.

Upon activation, IRE1 *α* oligomerises and autophosphorylates rendering its cytoplasmic kinase domain active. It has been proposed that the catalytic unit of active IRE1 is a dimer both in yeast and in mammals [13]. However, higher-order oligomers have also been detected *in vitro*[10,25,26]. According to this, we assume that the IRE1 *α* complex is formed of 4 monomers, and each quadromer has 2 catalytic domains. Each active domain catalyses the unconventional splicing of the XBP1 mRNA, which in turn translates into a transcription factor enhancing BiP synthesis. The ATF6 branch is connected to the IRE1 *α* branch through the regulation of XBP1 and BiP mRNA (Figure 4). The module comprises both fast-acting protein-level interactions and lengthy genetic regulatory interactions. In order to distinguish between these, we set the overall kinetics of receptor activation faster compared to the regulatory mechanisms that follow. In addition, we aim to obtain 3 to 4 times increase in BiP [27], and the splicing of a majority of the XBP1 mRNA in response to the activation of IRE1 *α*[23].

**Figure 5:**
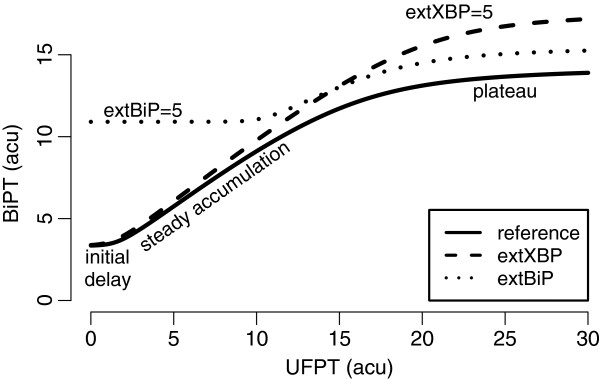
**The level of total BiP (BiPT) with increasing total UFP (UFPT) predicted by the IRE1 *α * module.** The black solid curve indicates the increase in the level of BiP when there is no external regulation (for the reference model see Additional file 1: Text 1.2). The dashed curve indicates the deviation from this when XBP1 mRNA is induced externally (extXBP), and the dotted curve indicates the change when the BiP mRNA is induced externally (extBiP) to the same degree.

The module responds to UFP accumulation with a steady elevation of BiP following a short delay caused by the cooperativity of IRE1 *α* activation (Figure 5). For moderate UFP levels, BiP production is nearly linear with respect to the receptor activation; however, it possesses an upper limit. That is, when the adaptive response falls short for managing the ER stress, and UFP accumulates to extreme levels, the pathway cannot provide additional BiP production; a plateau is reached.

Cleaved ATF6 is a transcription factor, which regulates the expression of both XBP1 and BiP [28,29], imposing external regulation to the IRE1 *α* branch. We observe that external regulation of BiP is primarily effective in elevating its basal levels for weak or no stress conditions. Since more BiP is available for concealing UFP, activation of the module is delayed, and we see an elongated initial lag-phase in Figure 5 (dotted curve). External regulation of XBP1, on the other hand, elevates the maximal BiP levels — higher plateau seen in Figure 5, dashed curve — allowing for the management of more severe stress conditions. However, there is an upper limit to BiP production, and the model suggests that external regulation on the IRE1 *α* branch is effective only to bring the folding capacity up to this limit (Additional file 1: Figure S3).

#### The ATF6 branch

**Figure 6:**
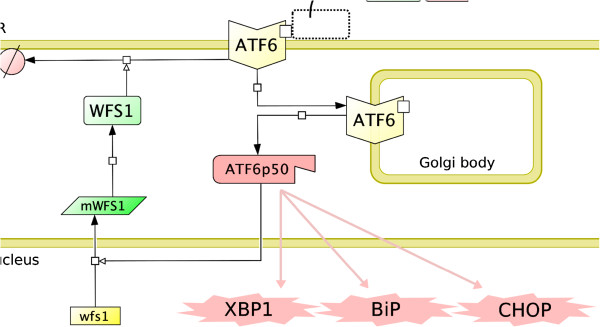
**The ATF6 branch of the adaptive response module.** The figure shows the three compartments involved in the mechanism of ATF6 activation. When BiP dissociates, the receptor is transported to the Golgi body as a monomer, and then it is cleaved by serine proteases. The cleaved domain, ATF6p50, acts as a transcription factor regulating the XBP1 branch and the translation attenuation module. It also activates WFS1, which controls the degradation of the receptor from the ER membrane.

We describe the ATF6 receptor as a monomeric transmembrane protein whose luminal ER excision site is hindered by BiP [12]. Following the accumulation of UFP, we assume the rate of translocation of the unbound ATF6 to the Golgi body depends linearly on receptor concentration. We incorporate the cleavage of its cytoplasmic domain as an implicit process whose overall kinetics is represented by a single rate constant parameter (*k*_cleave_ in Additional file 1: Text 1.3). The cleaved protein is a transcriptional activator of XBP1, BiP and CHOP linking adaptive response and translation attenuation modules together [12]. We also model the negative regulation of the receptor by WFS1, which is induced transcriptionally by the activated ATF6. In turn, WFS1 enhances the degradation of ATF6 on the ER membrane negatively regulating the UPR signalling [30,31] (Figure 6).

ATF6 has two isoforms, *α* and *β*, with different stabilities and activities [32]. For simplicity, we combine the two isoforms into a single entity, named conveniently as ATF6. We assume that the parameters controlling the attributes, *e.g.* synthesis and degradation rates, of ATF6 and its cleaved form are similar to those of IRE1 *α*. Tuning the remaining parameters, we match the basal ATF6 concentration approximately to that of IRE1 *α*. We set the rate of protein cleavage much higher than the rate of transfer in order to discriminate fast enzymatic reactions with slow membrane remodelling in consistency with the rest of the model. The ATF6 branch with its parameters configured accordingly can be seen in Additional file 1: Text 1.3.

**Figure 7:**
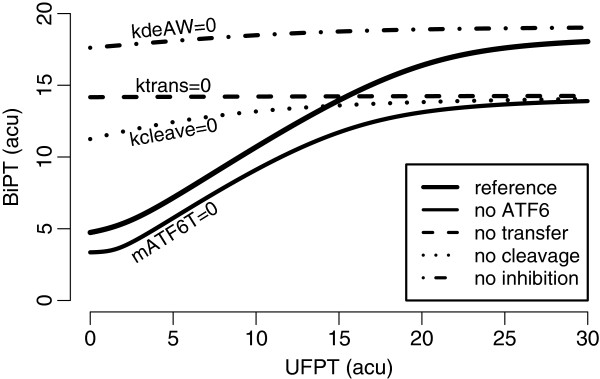
**The effect of the ATF6 branch on the adaptive response.** The figure shows the total BiP (BiPT) levels with respect to the total unfolded protein (UFPT). The thick black solid curve describes the change from the basal adaptive response, *i.e.* the thin solid curve, when ATF6 branch is involved. The dashed, dotted, and the hybrid curves, respectively, show the response when transfer or cleavage is inhibited, or when WFS1 is rendered ineffective. The reference model with both ATF6 and XBP1 branches is given in Additional file 1: Text 1.3.

As expected from the analysis of the IRE1 *α* module (Figure 5), we observe that ATF6 amplifies both the basal and the maximal folding capacity (Figure 7). In order to keep the basal folding capacity to a minimum level, we assume that ATF6 differentially contributes to the regulation of XBP1 and BiP. As a result, the impact in the active state becomes larger than that in the inactive state.

When the transfer of the unbound monomer is inhibited, we observe that the response stays unchanged; a high activation level is stably maintained even for no UFP. When the receptor accumulates excessively on the ER membrane, BiP shifts target from IRE1 *α*, and this results in the stable activation regardless of UFP. When only the cleavage is inhibited, a fraction of unbound ATF6 is transferred away from the ER relieving the early activation partially. However, both the ER membrane and the Golgi body will eventually become saturated with ATF6 resulting in the UFP-independent activation. Over-expression of ATF6 has experimentally been observed to enhance the UPR response regardless of the ER stress [33,34], which conforms with the model predictions, *i.e.* ATF6 might be able to divert BiP from IRE1 *α*.

We observe early activation and the independence on UFP also when the WFS1 regulation is inhibited. In this case, ATF6 accumulates in the ER and it is functional; therefore, BiP levels rise for all UFP concentrations. Elevation of BiP following the inhibition of WFS1 has also been experimentally observed [30,31].

As a result, the model of the ATF6 module suggests that the role of ATF6 in UPR activation is supplementary. The branch mainly regulates the basal and the maximal folding capacity assisting the adaptive response initiated by the IRE1 *α* branch.

#### The PERK branch - the translation attenuation module

The translation attenuation module is built around the phosphorylation cycle of the eukaryotic initiation factor eIF2 *α*[35]. The membrane receptor PERK is responsible for phosphorylating, thus deactivating, the initiation factor following the accumulation of UFP. When the active/unphosphorylated initiation factor levels drop below a certain threshold, 5’-cap dependent translation slows down substantially [36], but the translation of ATF4, CHOP and BiP selectively enhances [37-40]. Although CHOP is a well-known trigger for apoptosis, it is also known to activate GADD34, a phosphatase which alleviates the inhibition of eIF2 *α* and reactivates translation.

**Figure 8:**
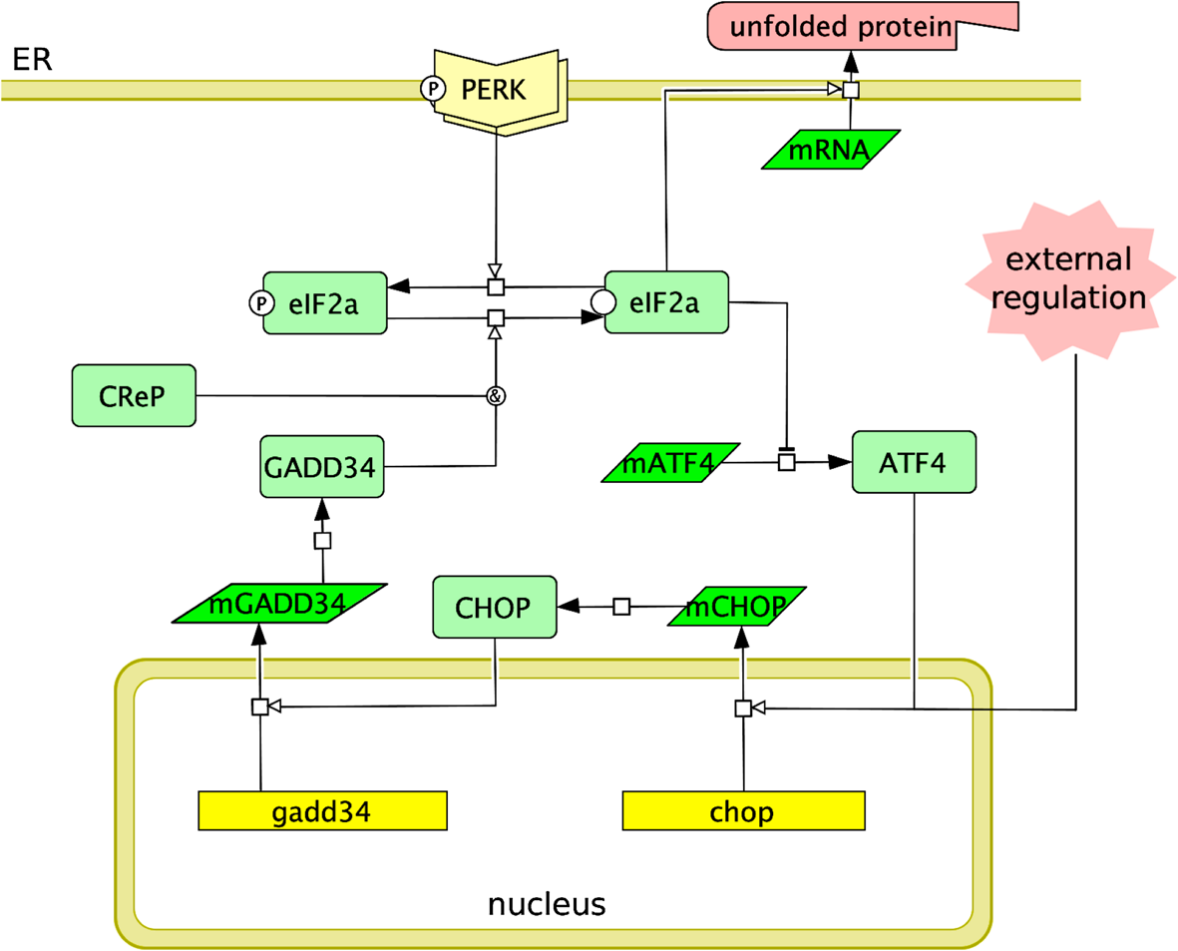
**PERK branch and the translation attenuation module.** The figure shows the post-translational and genetic regulatory steps involved in regulating translational rate in response to ER stress. Activated PERK phosphorylates and deactivates eIF2 *α*, which results in increased translation of ATF4. This triggers the activation of CHOP and subsequently GADD34, which negatively regulates eIF2 *α* phosphorylation and restores translation. Feedback provided by the ATF6 module is represented as external regulation on the expression of CHOP.

We condense most of the post-translational dynamics into the form of an ultrasensitive-switch [41,42], where activated PERK and phosphatases–GADD34 and CReP–compete for assessing the phosphorylation status of eIF2 *α*. The unphosphorylated eIF2 *α* suppresses the translation of ATF4 through an elaborate mechanism of ribosomal shift[43,44]. Adopting a black box approach, we interpret the mechanism with a Hill-type kinetics, where we assume about 90% decrease in active eIF2 *α* is sufficient to attenuate translation. To this basal translation attenuation mechanism, we incorporate genetic regulation of CHOP and GADD34, and also define the external regulation of CHOP by ATF6 and XBP1 [6,45] (Figure 8). The details of the module with corresponding parameterisation can be seen in Additional file 1: Text 1.4.1.

**Figure 9:**
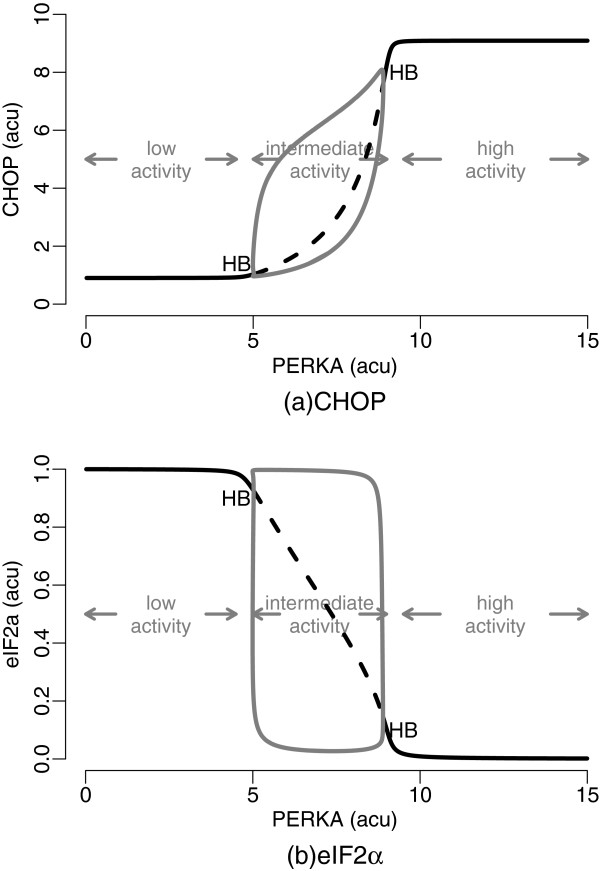
**Bifurcation diagrams showing the three activity states proposed by the translation attenuation module.** Solid dark curves indicate stable stationary points as they evolve through the three states in response to increasing levels of active PERK (PERKA). Dashed dark curves indicate unstable stationary points, and the solid grey curves indicate the minimum and maximum values of CHOP **(a)** and eIF2*α***(b)** oscillations. HB stands for Hopf bifurcation where a limit cycle transcends into dumped oscillations.

By analysing the bifurcation diagrams we detect three distinct states delineated by the activity of PERK (Figure 9). While the low activity state is characterised by low levels of CHOP and high translation rates, the high activity state is distinguished by high levels of CHOP and virtually suspended translation. It is evident from Figures 9A and 9B that the rate of translation and the expression of CHOP are complementary to each other. The states are essentially stable within wide windows of PERK activity; however, a pair of global bifurcation points, characterised as Hopf bifurcations, exists delineating the three activity states. These points lead to an interesting and *a priori* unexpected observation that an intermediate activity state exists, where many of the system components dynamically oscillate between low and high activity states.

We perform bifurcation analysis in order to determine the contribution of system parameters to the properties of the three states. We observe that a set of parameters adjusts CHOP levels for the low activity state, elevates the threshold for activation, and reduces the span of the intermediate state (Additional file 1: Figure S5). The set includes parameters responsible for directly controlling the activation dynamics of ATF4 (kATF4, nh and eIF2aT), and also the expression of the CHOP mRNA immediately downstream of ATF4 (extCHOP). In contrast, a set of parameters responsible for eIF2 *α* phosphorylation (CReP, kphos, kdephos and kmChop), results in a shift and a deformation in the intermediate state without effectively changing the levels of the low and high activity states (Additional file 1: Figure S6). Finally, the parameter kmAtff, which controls the potency of CHOP activation by ATF4, affects all of the three states changing the basal and the active response levels and also the span of the intermediate state (Additional file 1: Figure S6(d)).

In order to globally determine the range of parameter values responsible for oscillations, we employ a variation of the ABC-SMC algorithm [46]. That is, we use the following Heaviside step function as the distance measure,

$$H(x)=\left\{\begin{array}{cc} f\geq \epsilon & 1\\ f<\epsilon & 0 \end{array}\right),$$

where *f* stands for the frequency of oscillations, and *ϵ* is an arbitrary threshold. We raise *ϵ* for each generation; so that, in the final generation, we end up with a distribution of parameter values associated with high-frequency oscillations. The marginal distributions given in Additional file 1: Figure S7, demonstrate that high cooperativity in ATF4 activation (high nh) must be accompanied with low activation threshold (low kATF4), ample eIF2 *α* (high eIF2 *α*T), and minimal external regulation of phosphorylation (low CReP) and CHOP activation (low extCHOP) in order to maintain oscillations. The sensitivity matrix resulting from this distribution [47] indicates low sensitivity of the oscillation frequency against activated PERK, which is a strong indication of a broad intermediate activity state (Additional file 1: Figure S8).

In order to further investigate the origin of oscillations, we develop a reduced time-delay model describing the dynamics of ATF4 and GADD34 (Additional file 1: Text 1.4.2). Similar to the extended model, we observe the three activity states controlled by the level of active PERK (Additional file 1: Figure S9(a)). However, we observe oscillations in the intermediate state only when we introduce the time-delay resulting from the genetic interactions (Additional file 1: Figure S9(b)). The amplitude and period of the oscillations depend on the extent of this delay, *i.e.* the time it takes from the activation of ATF4 to the expression of GADD34 (Additional file 1: Figure S9(b) and S9(c)).

#### The BAX/BAK/BH3 pathway

In order to investigate the effect of UPR activation on the timing of apoptosis, we connect to the UPR model the mitochondrial BAX/BAK/BH3 apoptosis model of Zhang *et al.*[48]. For maintaining clarity and minimising the complexity of the ensemble, we use the condensed version described in Tyson *et al.* 2011 [49]. The equations and the list of parameters as used here is given in Additional file 1: Text 1.5 (Additional file 1: Table S3).

We connect the apoptosis module with the rest of the model by assuming that CHOP blocks the expression of Bcl-2 [35], which we describe with a Hill equation. We also assume that CHOP activates the transcription of Bim (BH3) [50], and replace the bifurcation parameter “Stress” with the concentration of CHOP. We preserve the parameters of the original model; however, introduce an additional set of parameters controlling the dynamics of Bcl-2 inhibition and Bim activation. In comparison to the bifurcation analysis presented in Tyson *et al.* 2011 [49], we present the behaviour of the pathway in response to varying CHOP levels in Additional file 1: Figure S9(a), and the time-dependent activation for a relatively high CHOP value in Additional file 1: Figure S9(b).

#### The reaction kinetics

The concentrations of both XBP1 mRNA and activated receptor complex can be low at some point during the activation, splicing or deactivation. For this reason, it might be inappropriate to use the Michaelis-Menten reaction kinetics as it stems from the assumptions that the enzyme concentration is fixed and the substrate concentration is greater than the enzyme concentration. Instead, we extend the Michaelis-Menten equation in order to accommodate variable concentrations of both enzyme and substrate. According to this, the equation for the rate of change in the concentration of the product, [*P*], can be written as

$$\begin{array}{ll} \;\frac{d}{\text{dt}}[P]=\frac{1}{2}k_{c} & \left(S_{t}+E_{t}+K_{m}\right)\\ & \left(-\sqrt{{\left(S_{t}+E_{t}+K_{m}\right)}^{2}-4\;S_{t}\;E_{t}}\right), \end{array}$$

where *k*_*c*_ is the maximum rate of catalysis, *K*_*m*_ is the “affinity” parameter, *i.e.* the amount of substrate needed for achieving half the maximum catalytic rate, and *S*_*t*_ and *E*_*t*_ are the total substrate and enzyme concentrations, respectively. Details of this derivation can be followed in the Additional file 1: Text 2.

For the downstream genetic regulatory interactions of the UPR, we require a generic regulatory model, which could accommodate many effectors acting on a single copy of a gene. Using the well-established models of genetic regulation [51], we derive the following equation describing the rate of change in mRNA concentration:

$$\frac{d}{\text{dt}}[\text{mRNA}]=\frac{\underset{i}{\sum }\;k_{\text{ci}}\;{\left[\text{TF}\right]}_{i}/K_{m_{i}}}{1+\underset{j}{\sum }\;{\left[\text{TF}\right]}_{j}/K_{m_{j}}},$$

where *i* denotes the *i*^*th*^ element in the set of transcriptional activators, and *j* denotes the *j*^*th*^ element among all regulators. In the equation, [TF] is the concentration of a transcription regulator, *k*_*c*_ is the maximum rate of activation, and *K*_*m*_ is the relative affinity of the TF to the gene. The model assumes competing transcription factors enhancing/diminishing the transcription of a single or a low-copy gene. Details of the derivation can be followed in the Additional file 1: Text 3.

This is, of course, only a first-order approximation of the underlying dynamics assuming adiabatic decoupling of transcription and its regulation. This assumption can be relaxed, and the model of regulation can readily be improved upon if desired for further studies. In this context, as seen in Additional file 1: Table S2, we use this equation for the regulation of XBP1, BiP, WFS1, ATF4, CHOP and GADD34.

### The three distinct activation patterns of the UPR

The complete model of the UPR includes all four of the functional modules as well as the apoptosis module composed into a single coherent system. In order to facilitate the conduction of stress signals, initially in the form of receptor activation, we tune certain key parameters to match the range of signals required to activate a downstream pathway to that which is delivered by its immediate upstream neighbour. These parameters include the rate of eIF2 *α* phosphorylation and dephosphorylation, with which we enable translation attenuation to conform the range of PERK activation. In addition, the CHOP-associated parameters of the apoptosis module, for instance, help to juxtapose the intermediate activation states of CHOP and BAX. The complete model with the working parameter set is listed in Additional file 1: Table S1 and Table S2.

**Figure 10:**
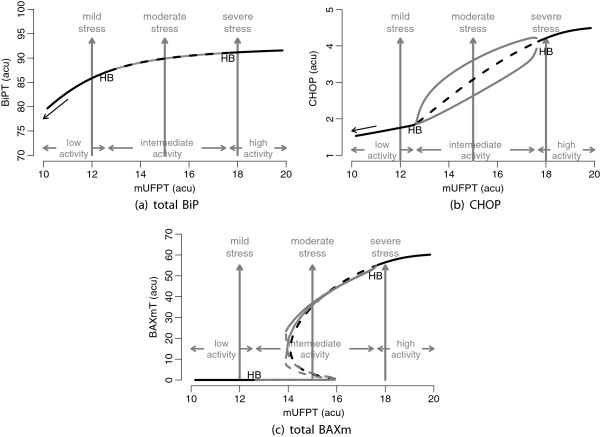
**Bifurcation diagrams showing the behaviour of the complete UPR model for various stress levels.** The boundaries of the three activity states are also shown with horizontal arrows. The black arrows in **(a)** and **(b)** indicate the trend for milder stress conditions which are omitted from the plots. Three of the stress conditions have been marked as mild, moderate and severe for use in the analyses. These correspond to the values 12, 15 and 18, respectively, for the rate of total unfolded protein accumulation (mUFPT). The diagrams are plotted as in Figure 9.

As a result, we observe a steady accumulation of adaptive measures, raising BiP reserves in Figure 10(a), prior to the intermediate activity state for mild stress conditions. When stress conditions aggravate, oscillations commence, prevalent in CHOP and the rate of translation (Figure 10b), and eventually the system encounters a bistable apoptotic switch shown in Figure 10(c). For most of the intermediate state, *i.e.* for moderate stress conditions, localisation and activation of BAX are suppressed. However, the high activity state with elevated apoptotic signals is reached upon breaching the threshold. Perceiving the outcome of the intermediate state is not straightforward given that the behaviour depends heavily on from where the state has been reached. For instance, attempts to reverse the maladaptive response by reducing the rate of UFP accumulation will require a greater effort compared to what is needed to drive the system off the threshold. What is responsible for this difference is the extent of the bistable region of the apoptotic module. We will address this characteristic of the model further following the investigation of model validity.

### Predictions agree with experimental observations

**Figure 11:**
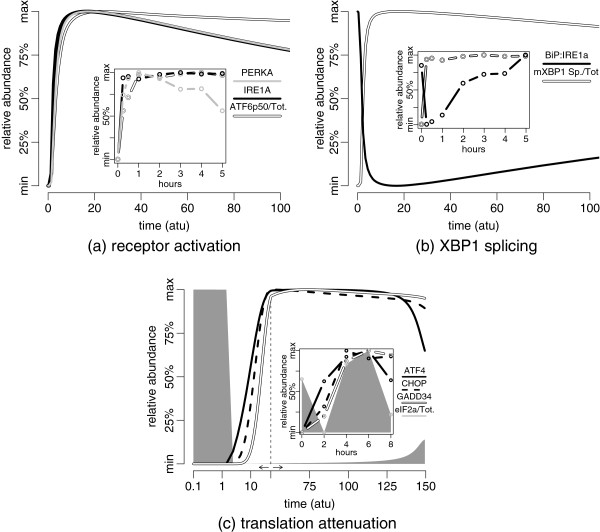
**Activation of the UPR following the accumulation of UFP in the ER.** Synchronised activation of the three receptors, IRE1 *α*, ATF6 and PERK, is shown in **(a)**. Splicing of the XBP1 mRNA and the displacement of BiP from IRE1 *α* are shown in **(b)**. Relative timing of translation attenuation and the signal transduction through the PERK branch are plotted in **(c)**. The time axis in **(c)** for early activation is given in log-scale for improved visual discrimination. The analogous experimental observations as adapted from Figure 8B of DuRose *et al.* 2006 [23]**(a)**, Figure 7C of DuRose *et al.* 2006 [23]**(b)** and Figure 3A of Marciniak *et al.* 2004 [53]**(c)** are given in the insets of the figures. The level of phosphorylated receptor is calculated by multiplying the activated IRE1 *α* and PERK with the stoichiometry of the activated complex. The percentage of cleaved ATF6 is calculated with respect to the amount of ATF6 on the ER membrane and Golgi body together with cleaved ATF6. In order to facilitate the effective comparison of partial and total activation levels of different components, both simulations and data are re-normalised to 0-100% of their respective ranges. As a result, the 50% mark along the y-axis represents the median of the range of values observed from 0 to 100 *atu* (or 150 *atu* for (c)) for each component. The parameters used for the simulations are given in Additional file 1: Table S2. The moderate stress condition is chosen with reference to Figure 10. The experimental data presented are extracted from the respective publications with the aim of aiding visual comparison. The reader is referred to these publications for the original reports of the data.

In order to allow justification for model predictions with regards to previously observed experimental data, we simulate the response against an arbitrary stress condition — moderate is appropriate in this context — and follow the initiation and the transmission of stress signals. As a result, we observe simultaneous activation of IRE1 *α* and PERK, as well as the cleavage of ATF6 upon UFP accumulation (Figure 11(a)). Following the rapid activation, we observe a slow but steady decrease in the activated IRE1 *α* and PERK, which is associated with the gradual accumulation of BiP as a result of the initiation of the adaptive response. ATF6 cleavage appears to be more resistant to being tuned down due to the differences in its mechanism of activation; namely, low cooperativity and the requirement to be replenished by the newly synthesised receptors.

We observe a similar trend in an exemplar experimental observation by DuRose *et al.*[23]. Their observations, as transformed likewise as our predictions for comparison, are given in the inset of Figure 11(a). There, we also see the reduction in the activation of PERK as a signature of model validity. The main difference is the trend of IRE1 *α* activation, which is closer in relation to that of ATF6 rather than that of PERK. This difference could stimulate experimental studies on the differences between the activation dynamics of IRE1 *α* and PERK. However, according to the model, it could tentatively be explained by a stronger association coefficient of the IRE1 *α* complex.

Further to receptor activation, displacement of BiP from the receptors and its gradual re-association into the BiP-receptor heterodimer can be seen in Figure 11b. The level of receptor activation is sufficient, as expected, to trigger the splicing of the XBP1 mRNA, and the phosphorylation of eIF2 *α*. Also in this case, the experimental observations of DuRose *et al.*[23] (given in the inset of Figure 11(b) as transformed accordingly) abide well with the model predictions. We observe the characteristic displacement and subsequent gradual replacement of BiP on the receptors; however, the extent of recovery of BiP-bound receptors are predicted lower than the observed. DuRose *et al.* addressed this issue arguing that the observed BiP-bound receptors were more than expected due to the possibility of BiP binding to the phosphorylated/active receptors [23].

According to the model, BiP is primarily responsible for tuning down the UPR activation and resuming the rate of translation despite unmitigated stress conditions. That is, accumulating BiP gradually sequesters the receptors and, acting as a negative regulator, diminishes the level of active membrane receptor. This results in a reduction of activated PERK, which prevents the system from attaining the high activity state for long. The inhibitory effect of BiP has also been reported previously both in experimental [20-23] and theoretical [14,52] studies.

The model facilitates the investigation of the translation attenuation kinetics in the course of developing adaptive response. As seen in Figure 11(c), activation of ATF4 begins shortly after eIF2 *α* is phosphorylated. As expected, the immediate response to UFP accumulation is translation attenuation owing to its entirely post-translational kinetics. Following the activation of ATF4, CHOP and GADD34 get activated, and they act together on eIF2 *α*. With the current configuration of parameters, GADD34 is not potent enough to quickly reactivate translation, but it requires BiP to accumulate and weaken, indirectly though PERK and CHOP, the rate of phosphorylation of eIF2 *α*. Though the end of the time-course in Figure 11(c), we begin to observe the consequences of accumulating BiP.

A similar experimental observation was published by Marciniak *et al.*[53] (given in the inset of Figure 11(c) as transformed accordingly). There we see also the sequential activation of ATF4, CHOP and GADD34, as well as the immediate translational response and recovery. Although the recovery of translation is too rapid compared to the predictions, we have already shown in the comprehensive analysis of the PERK branch that increasing the rate of dephosphorylation by GADD34 or decreasing the rate of phosphorylation by CHOP (Additional file 1: Figure S6(b) and S6(c)) will be sufficient to shift the intermediate activity range. This, in turn, enables an accelerated recovery of translation attenuation as observed.

The transient nature of eIF2 *α* phosphorylation has previously been reported [54,55], where it was primarily attributed to the inhibitory effect of GADD34 activation. However, due to the inhibitory effect of BiP, the model suggests that the system might eventually traverse back to the intermediate state where oscillations in translation persist. In Figure 11(c), we barely observe the resuming of translation due to the slow build up of BiP; however, in the following section we investigate further the consequences of the long-term activation of the UPR as predicted by the model.

### Preconditioning acts by developing adaptation and tolerance

**Figure 12:**
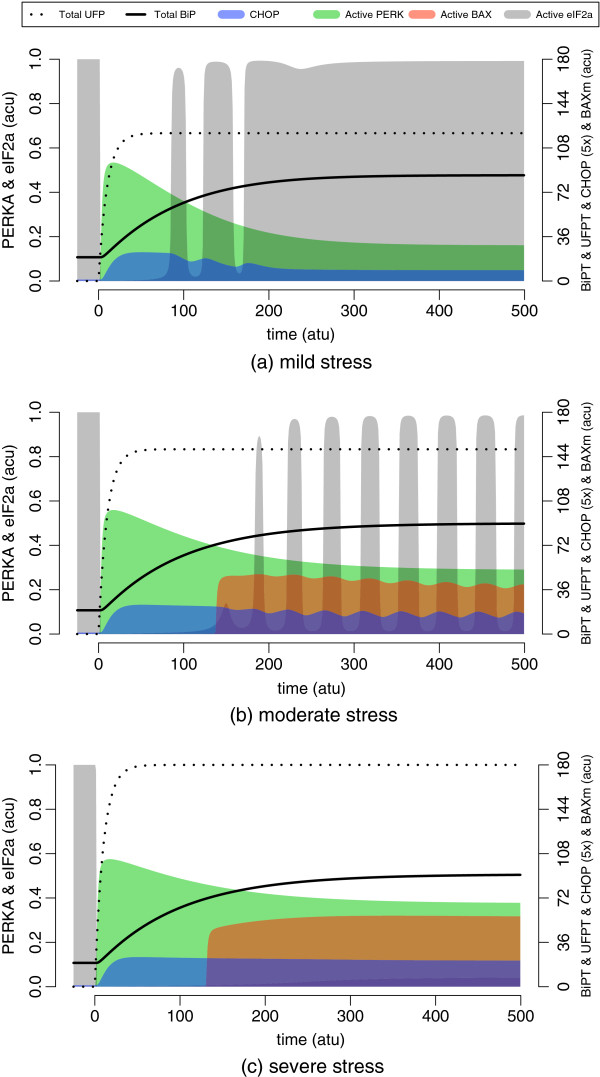
**The low, intermediate and high activity states of the UPR.** The low activity state is shown in **(a)**, where rapid but transient activation of CHOP is followed by the recovery of translational activity. In **(b)**, the intermediate state is shown, which exhibits sustained oscillations in both CHOP and the rate of translation. This eventually leads to the activation of BAX on mitochondrial membrane. In **(c)**, the high activity state is shown with elevated and sustained UPR activity, *i.e.* activation of PERK and expression of CHOP. The plot also shows severely reduced translation rates and the activation of apoptotic signals. The legend is given on top of the plots. The grey shades indicate active eIF2 *α*, which represents the relative rate of translation. The initial conditions have been extended towards the negative time axis in order to demonstrate the punctuality of the translational response. The stress conditions chosen are based on Figure 10.

In order to demonstrate better the three phases of translation attenuation we simulate the model with mild, moderate and severe stress conditions as marked previously in Figure 10. We observe, in each case, that the primary response of the pathway is to turn down the rate of translation immediately (Figure 12). Since this is controlled by direct protein-protein interactions, and it is immediately downstream of PERK, translational response takes place before the elevation of folding capacity as expected.

The system initially possesses low folding capacity; therefore, it assumes the high activity state upon activation. It attenuates translation, elevates CHOP, and in the mean time, activates the adaptive response, *i.e.* chaperone synthesis. In this context, we concentrate on unmitigated stress conditions where the ER stays irresponsive against the UPR outcome. Despite this, we observe the accumulation of BiP suppressing the activation in time. Suppression, in turn, might result in resumption of translation, reduction of CHOP levels and aberration of apoptotic response. Effectiveness of the folding capacity, BiP accumulation in particular, determines whether the maladaptive response can be avoided or not in cases of sustained ER stress. For instance, for mild (Figure 12(a) and moderate (Figure 12(b) stress conditions, we observe that the adaptive response manages to divert the outcome to low and intermediate activity states, respectively.

Here, it is worth noting that the low activity state is characterised both by the level of UFP and the elevated folding capacity, *i.e.* BiP levels, in the ER. The resulting behaviour can be interpreted as stress adaptation, where sufficient BiP is available to suppress UPR and the maladaptive response. However, in the case of severe stress (Figure 12(c), BiP fails to cope with extreme UFP, and also, to suppress UPR activation. Therefore, the adaptive response is averted and it is replaced by a strong commitment to apoptosis.

**Figure 13:**
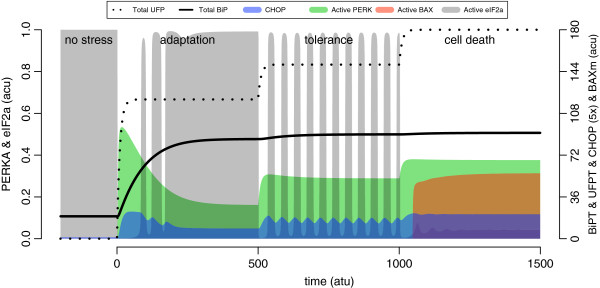
**The UPR response against stepwise escalation of the ER stress levels.** The mild, moderate and severe stress conditions as in Figure 12 are administered sequentially for a duration of 500 time units each. The elevation of active PERK, BiP and CHOP, the status of translation, and the activation of BAX on mitochondrial membrane are shown with respect to accumulating UFP. The grey shade indicates active eIF2 *α*, which represents the relative rate of translation. The initial conditions have been extended towards the negative time axis in order to demonstrate the punctuality of the translational response. The plot is drawn as in Figure 12, and the legend is given on top of the plot.

This is problematic for the ER, as seen in Figure 12(b); because, accumulation of BiP switches the response to the intermediate activity state but from the high activity state. As a consequence, the threshold for apoptosis has already been breached, and the switch becomes inefficient to annihilate the maladaptive response — BAX is activated for apoptosis to commence. It is, however, possible to reach to the intermediate state from the low activity state by first introducing a mild stress and letting the system attain the adaptive phase with elevated folding capacity. In this case, the apoptosis module resides at the off state, and remains there despite the elevation of CHOP and the appearance of oscillations (Figure 13).

According to the model, the limit to the folding capacity of the ER is a major determinant of the initiation of apoptosis. When the limit is reached, as seen in the rightmost panel of Figure 13, no more BiP can be expressed to suppress the UPR further. Therefore, any additional increase in the level of CHOP can easily bring the system above the apoptotic threshold. Folding capacity could be enhanced by specifically inducing BiP, or inflicting a mild but sustained stress condition with a chemical agent. Mild stress results in the development of an adaptive phase during which UFP and BiP levels rise in the ER, and this is protective against aggravating stress. Therefore, the protective power of preconditioning, according to the model, is limited to the maximum level of BiP a cell is capable of expressing.

### Oscillations control cellular activity during stress adaptation

Prior to conclusion, we examine the possible implications of oscillatory behaviour. We have already shown that the difference in the time scales of eIF2 *α* turnover and the inflicted genetic regulations results in the appearance of oscillations in many system components, *e.g.* CHOP and the rate of translation, for moderate stress conditions. If this difference is minimised, for instance by reducing the rate of eIF2 *α* phosphorylation and dephosphorylation, we expect to observe a smooth gradual transition through the intermediate state. This, in turn, results in a complete attenuation of translation for moderate stress conditions as seen in Additional file 1: Figure S11(b), as opposed to minor changes seen for mild and severe conditions (Additional file 1: Figure S11(a) and S11(c) in Additional file 1: Text 1.6).

On the other hand, when the stress conditions progressively worsen, we observe that the low and high activity states change nominally; however, translation becomes permanently attenuated upon entry to the intermediate activity state (Additional file 1: Figure S11(d)). We have deemed such a transition in Figure 13 as the development of stress toleration, where translation resumes at least for some periods. It is, however, more appropriate to consider the non-oscillatory intermediate state as senescence, because of the lack of translation seen together with no apoptotic activity. As a result, the model suggests that the existence of oscillations provides a means for translation, and hence the routine cellular activity, to be partially restored.

### Discussion

The UPR is composed of a complicated mesh of biochemical and genetic regulatory interactions. These range from unconventional mRNA splicing, global translational disruption and the activation of hundreds of genes with a single aim to deliver the right response at the right time [56]. The decision and timing of an appropriate response are implemented within the intricate wiring of this signalling cascade, which we aimed to decipher by constructing its detailed mechanistic model. The model incorporated the three main signalling pathways, *i.e.* IRE1 *α*, PERK and ATF6, the interconnections between these pathways, and the downstream genetic regulatory interactions. To the best of our knowledge, this model is the first in its extent and in the detail it incorporates.

To day, there have been two major approaches to the modelling of the UPR, but each of these focused on a specific part of the cascade. The model of Pincus *et al.*[14] was one of the first studies to justify the involvement of BiP in regulating the UPR activation. However, it only incorporated the proposed mechanism of yeast IRE1 activation. The translation attenuation model of Trusina *et al.*[52,57], described the overall dynamics of IRE1 *α* and PERK with an emphasis to the relative effects of chaperone synthesis and translation attenuation on alleviating the ER stress. Rutkowski *et al.*[58] had also developed a simple UPR model in order to explain the transient activation of CHOP and GADD34 even in cases of weak ER stress. They observed the adaptive behaviour of the UPR and suggested that the factor responsible for it might be the differential stabilities of chaperones compared to the other UPR components. The model of the UPR we have developed, assembles a larger more detailed version of the UPR, and in addition to confirming the findings from these previous models, it proposes a plausible decision mechanism for the initiation and timing of apoptosis.

One major prediction of the model is the existence of three identifiable states of behaviour the UPR might exhibit. An appropriate behaviour is computed and executed depending on the level and duration of stress, and also the availability of BiP. The low activity state, to begin with, is characterised with the ability to elevate folding capacity. At this stage, the effort is focused on the elevation of BiP, assisting protein folding and preventing further activation of the UPR.

According to the model, BiP can act both as a positive regulator and as a negative regulator of the UPR by switching between the receptors and UFP. This assigns the chaperone a pivotal role during the low activity state where it helps to coordinate the development of stress adaptation. BiP has previously been associated experimentally with adaptation [21,58-60], which we predict to occur when sufficient chaperone accumulates to suppress UPR signalling and prevent the elevation of CHOP, the signal for apoptosis.

Adaptation is compromised when the limit of chaperone synthesis is reached. For severe stress conditions, this results in the elevation of apoptotic signals and the irreversible activation of the BAX/BAK/BH3 pathway. At this stage, the rate of translation is sustained at a minimum level, which might be unfavourable for apoptotic activity due to the inability to synthesise certain proteins [61,62]. We speculate that direct binding of BAX (and BAK) to IRE1 *α*[63] on the ER membrane may be essential to activate an alternative pathway, for instance the JNK pathway and the unspecific mRNA decay mechanism [24]. This in turn may promote apoptosis especially when it is augmented with the disruption of the Ca ^+2^ balance — caused by the activated BAX.

The model predicts an intermediate activity state during which CHOP is activated but has yet to reach its upper limit. During this state, we observed oscillations in many system components, including the rate of translation, for the first time to our knowledge. Oscillations occur as a result of differences in the kinetics of eIF2 *α* phosphorylation/dephosphorylation and genetic regulation, and this plays a crucial role in resuming translation at least for brief periods of time. We speculate that translation at this stage might be beneficial in the continuation of vital cellular functions, or, especially if the apoptosis is initiated, in the synthesis of apoptotic genes.

The current configuration of the model parameters permits the alignment of the intermediate activity region of CHOP with the bistable range of BAX. As CHOP levels raise the system moves across the bistable regime exceeding the activation threshold just before CHOP reaches its upper limit. Therefore, the maladaptive behaviour at the intermediate state depends heavily on from where it is reached. For instance, applying enough stress to bring the system to the intermediate activity state from an unstressed ER will cause the elevation of apoptotic signals. This is mainly because of the shortage of time for BiP to accumulate to suppress UPR signalling, leading to the appearance of first the high and then the intermediate activity state. Here, the importance of existence of an early stage of adaptation becomes obvious. Developing adaptation, or preconditioning in clinical terms [5,8,59,64,65], enables the elevation of folding capacity, and BiP, resting the system at the low activity state. When the intermediate state is reached from there, BAX remains low at the inactive branch of the bistable regime providing protection from apoptosis.

Regardless of where it is reached from the intermediate state exhibits oscillations in system components. However, they can be exhausted if the time difference between the phosphorylation of eIF2 *α* and the activation of GADD34 is reduced. By doing so, we noticed that the major contribution of oscillatory behaviour to the outcome of the UPR is the resuming of translational activity. In the no-oscillation case, during the intermediate state, there is absolutely no translational activity upon UPR activation. Moreover, if the system resides on the inactive branch of the apoptotic switch, in addition to translation attenuation, the activation of BAX will be permanently suppressed. It is only natural to expect this state of senescence to end shortly due to the gradual degradation of critical cellular functions.

We hypothesise that senescence and apoptosis might be preferred or avoided depending on the cell type. For instance, some of the vital cell types that cannot be replaced when damaged, *e.g.* nerve cells or podocytes of kidney, might be adapted to exhibit oscillations so that translation is resumed in part as a survival response. On the other hand, it might be beneficial for a lymphocyte to self-destruct promptly in case of any malevolent consequences of cellular damage. Testing the validity of this hypothesis, however, extends beyond the intended scope of this research.

The precise mechanisms of receptor dynamics, genetic regulation and crosstalk with other stress signalling pathways are currently unknown. This contributes greatly to the inevitable incompleteness of modelling approaches alike. However, with this research, we presented a mathematical model, which is, being faithful to the existing literature, highly predictive despite the absence of a perfect quantitative match between the predictions and the experimental observations. The modular step-by-step approach of constructing the model has been a major factor in easing the analysis and supplying this predictive power. The choice of the parameter values originated from the bifurcation analyses with reference to the experimental observations from literature. As experimental observations accumulate, the inaccuracies and disagreements between the predictions and the observations will form a strong basis for improving and extending this model. Consequently, such studies will necessitate the accommodation of data variability in terms of intrinsic stochastic fluctuations of the system. In order to address this issue, we are currently working towards relaxing the deterministic assumption and studying the three types of UPR output under the influence of intrinsic noise.

Nevertheless, a particular configuration of parameters might be valid for a certain cell type under certain extra- or intracellular conditions at a specific developmental stage. We argue that it is possible to tune the model of the UPR to represent the signalling cascade during most of such specific conditions. Consequently, the model should yield a response similar to what has been investigated in this work. An important step towards the validation of the model predictions, is to design a titration experiment where the ER is subjected to different stress conditions and the formation of the three distinct types of behaviour is observed: the low activity state with adaptive behaviour, the intermediate activity state with oscillations and bistability in apoptotic signals, and the high activity state with strong commitment to apoptosis.

An interesting experimental challenge as a natural consequence of this research would be to look for modifier genes in the UPR for related diseases. It might be possible that, for instance, any mutation or malfunctioning resulting in the manipulation of the intermediate activity state results in adopting the high activity state prematurely. In this case, translation may be attenuated and apoptotic signals elevated even though the ER stress is mild or moderate. In light of this, one of the major undertakings of our group is currently the investigation of the contribution of the UPR to the vast phenotypic heterogeneity of Alport Syndrome and Thin Basement Membrane Nephropathy [1,66].

## Conclusion

Here we develop, for the first time, a combined mechanistic model of the three signalling pathways of the UPR cascade. The model incorporates highly detailed enzymatic and genetic regulatory interactions based on the recent literature. The analysis of the model reveals that the balance between the ER stress and the folding capacity of the ER plays a pivotal role in managing the transformation from an adaptive to a maladaptive response. According to this, there exists three distinct states of behaviour the UPR may adopt: low, intermediate and high activity states. We demonstrate, for the first time, that under the right circumstances, the intermediate state may exhibit oscillations in translation attenuation and apoptotic signals. Demonstration of stress adaptation provides a mechanistic explanation as to how preconditioning might prevent the initiation of apoptosis. The model can be configured to represent the UPR of a specific cell type under certain experimental conditions. The experimental validation of the model predictions is currently one of the major undertakings of our group.

## Methods

The complete list of differential equations, derivations of reaction kinetics, and the choice of parameter values are explained in detail in the Additional file 1. The SBMLv2.4 version of the model is submitted to the BioModels Database [67] with the identifier BIOMD0000000446. The bifurcation analysis of the model is performed with XPPAUT5.41. The wiring diagrams are created in CellDesigner^TM^[68].

## Abbreviations

UFP: unfolded protein; UFPT: total UFP; BiP/GRP78: immunoglobulin binding protein / glucose regulated protein; IRE1α: inositol requiring protein 1α; PERK: protein kinase RNA-like ER kinase; ATF6: activating transcription factor 6; ATF4: activating transcription factor 4; GADD34: growth arrest and DNA damage-34; CHOP/GADD153: CCAAT/enhancer-binding protein homologous protein; XBP1: X-box binding protein 1; eIF2α: eukaryotic initiation factor 2α; CReP: constitutive repressor of eIF2α phosphorylation.

## Competing interests

The authors declare that they have no competing interests.

## Authors’ contributions

KE, MP and CD conceived the project. KE designed the study, developed the model and performed the analysis. KE and CD contributed to the final version of the manuscript. All authors read and approved the final manuscript.

## Supplementary Material

Additional file 1 **Supplementary Text.** The Supplementary Text includes detailed technical information about the mathematical model, its assumptions and supplementary analyses to the main manuscript concerning the effects of a broader range of the model parameters. Click here for file
