# Supplementary material for: A mathematical model of the unfolded protein stress response reveals the decision mechanism for recovery, adaptation and apoptosis
Source: BMC Syst Biol. 2013 Feb 21;7:16. doi: 10.1186/1752-0509-7-16 (PMC3695880; doi:10.1186/1752-0509-7-16)
Supplement: Additional file 1 — Supplementary Text. The Supplementary Text includes detailed technical information about the mathematical model, its assumptions and supplementary analyses to the main manuscript concerning the effects of a broader range of the model parameters. [file 1752-0509-7-16-S1.pdf]

Supplementary Text

A mathematical model of the unfolded protein stress response reveals the decision mechanism for recovery, adaptation and apoptosis

Kamil Erguler[a], Myrtani Pieri[a], Constantinos Deltas[a]

1 The UPR Model

The main assumptions of the model could be written as follows:

- We ignore the regulation of the maladaptive response through tumour necrosis factor receptor (TNFR)-associated factor-2 (TRAF2) and Jun N-terminal kinase (JNK) response elements.
- Since post-translational modification take place at a faster pace than genetic regulations [1], we assume they attain a quasi-steady-state within the time frame of genetic interactions.
- We assume that the unfolded proteins degrade at a constant rate directed by housekeeping mechanisms and ERAD, and this rate does not change with the activation of the UPR.
- We assume the translation attenuation only affects the translation rate of proteins directed into the ER, but not the components of the UPR pathway [2].
- For maintaining clarity, we assume that BiP binds to unfolded proteins as strongly as it binds to the ER stress receptors, and also as the receptors bind to each other to form homodimers/oligomers.
- We assume the stoichiometries of associated IRE1α and PERK, given by n, are the same [3].
- We assume fast kinetics of solute transfer between or within compartments within the time frame of genetic regulation.
- We assume cleaved ATF6 acts as a transcription factor to enhance the transcription of XBP1, BiP and CHOP mRNA [4, 5]. BiP and CHOP are upregulated through the ER stress response element (ERSE) promoter, which can be enhanced either by ATF6 or XBP1, or both [6]. ATF6 has also been shown to enhance XBP1 transcription upon ER stress [7, 3, 5].
- We assume that ATF4 not only activates CHOP, but also enhances the transcription of XBP1 mRNA [8, 3].

[0a] *Molecular Medicine Research Center and Laboratory of Molecular and Medical Genetics, Department of Biological Sciences, University of Cyprus, Kallipoleos 75, 1678 Nicosia, Cyprus*

- For maintaining clarity, we assume the concentrations of ATF4 and ATF6 mRNA are fixed.
- We assume that XBP1 and BiP mRNA have constitutive enhancers, which we represent with parameters "basalXBP" and "basalBiP", respectively, for maintaining a basal transcription rate.
- We assume that the steady-state levels of IRE1α, PERK and ATF6 in an unstressed ER are close to each other.

Table S1: Species and parameters in the UPR model given in terms of arbitrary time units (*atu*) and arbitrary concentration units (*acu*).

Abbreviation	Description	Unit	Value / Initial
[UFPT]	unfolded protein concentration	*acu*	0
[mUFPT]	pool of mRNA destined for being translated into the ER, which eventually end up not being properly folded	*acu*	0
[eIF2a]	eukaryotic initiation factor (active)	*acu*	0
[eIF2aT]	total eukaryotic initiation factor	*acu*	1
[BiP · UFP]	BiP bound to an unfolded protein	*acu*	0
[BiP]	concentration of BiP in ER lumen	*acu*	0
[BiPT]	total concentration of BiP in the ER including the proteins in complex	*acu*	0
[BiP · IRE1α]	BiP bound to an inactive IRE1α monomer	*acu*	0
[IRE1α]	concentration of IRE1α	*acu*	0
[IRE1αT]	total IRE1α concentration including the receptors in complex	*acu*	1
[IRE1αA]	activated, *i.e.* oligomerised and phosphorylated, IRE1α	*acu*	0
[BiP · PERK]	BiP bound to an inactive PERK monomer	*acu*	0
[PERK]	concentration of PERK	*acu*	0
[PERKT]	total PERK concentration including the receptors in complex	*acu*	1
[PERKA]	activated, *i.e.* oligomerised and phosphorylated, PERK	*acu*	0
[BiP · ATF6]	BiP bound to an inactive ATF6 monomer	*acu*	0
[ATF6]	concentration of ATF6	*acu*	0
[ATF6T]	total ATF6 concentration including the receptors in complex	*acu*	0
[mXbp1u]	unspliced XBP1 mRNA concentration	*acu*	0
[mXbp1s]	spliced XBP1 mRNA concentration	*acu*	0
[Xbp1s]	XBP1 protein translated from the spliced mRNA	*acu*	0
[mBiPT]	BiP mRNA concentration	*acu*	0
[mATF6T]	ATF6 mRNA concentration in cytoplasm	*acu*	5
[ATF6p50]	cleaved cytoplasmic domain of ATF6	*acu*	0
[ATF6T]	concentration of ATF6 residing on the ER membrane	*acu*	0
[ATF6GB]	concentration of ATF6 being transported to the Golgi membrane	*acu*	0
[ATF6p50]	concentration of cleaved ATF6 in the cytoplasm	*acu*	0

Table S1: Species and parameters in the UPR model given in terms of arbitrary time units (*atu*) and arbitrary concentration units (*acu*).

Abbreviation	Description	Unit	Value / Initial
[mWFS1]	Wolfram syndrome 1 (WFS1) mRNA concentration	*acu*	0
[WFS1]	WFS1 protein concentration	*acu*	0
[mATF4]	ATF4 mRNA concentration in cytoplasm	*acu*	1
[ATF4]	ATF4 protein concentration	*acu*	0
[CReP]	CReP concentration	*acu*	0.1
[mCHOP]	CHOP mRNA concentration	*acu*	0
[CHOP]	CHOP protein concentration	*acu*	0
[mGADD34]	GADD34 mRNA concentration	*acu*	0
[GADD34]	GADD34 protein concentration	*acu*	0
kf	rate of association for UFP, BiP, and membrane receptors	$acu^{-1}\,atu^{-1}$ or $acu^{1-n}\,atu^{-1}$	100
kr	rate of dissociation for UFP, BiP, and membrane receptors	atu^{-1}	10
n	stoichiometry of oligomerised and activated IRE1α and PERK ($n \in \{1, 2, 3, \dots\}$)	·	4
nh	cooperativity for ATF4 induction by eIF2α	·	2
basalXBP	concentration of a constitutive enhancer of XBP1 mRNA transcription	*acu*	1
basalBiP	concentration of a constitutive enhancer of BiP mRNA transcription	*acu*	1
krcXU	concentration of transcription factor necessary to transcribe XBP1 half the maximum rate	*acu*	5
krcBiP	reference affinity towards the promoter region of the BiP gene	*acu*	5
krcWFS	concentration of cleaved ATF6 required to achieve half the maximum transcription rate	*acu*	1
krcCHOP	concentration of ATF4 and other enhancers required for half the maximum rate of CHOP transcription	*acu*	1
krcGADD34	concentration of CHOP required for half the maximum rate of GADD34 transcription	*acu*	1
kmXbp	contribution of XBP1s to the transcription of BiP mRNA	·	10
kmAtfsXBP	contribution of ATF6p50 to the transcription of XBP1 mRNA	·	10
kmAtfsBiP	contribution of ATF6p50 to the transcription of BiP mRNA	·	1
kmAtff	contribution of ATF4 to the transcription of CHOP mRNA	·	0.05
kmChop	contribution of CHOP to the transcription of GADD34 mRNA	·	0.05
kmAtfs	contribution of ATF6p50 to the transcription of CHOP mRNA	·	0.1
ksplice	maximum rate of XBP1 mRNA splicing	atu^{-1}	10

Table S1: Species and parameters in the UPR model given in terms of arbitrary time units (atu) and arbitrary concentration units (acu).

Abbreviation	Description	Unit	Value / Initial
krcSplice	concentrations of substrate and enzyme required for achieving half the maximum rate	acu	1
trcXU	maximum rate of XBP1 mRNA translation	$acu\, atu^{-1}$	1
trcBiP	maximum rate of BiP mRNA transcription	$acu\, atu^{-1}$	1
trcWFS	maximum rate of WFS1 mRNA transcription	$acu\, atu^{-1}$	1
trcCHOP	maximum rate of transcription of CHOP mRNA	$acu\, atu^{-1}$	1
trcGADD34	maximum rate of GADD34 mRNA transcription	$acu\, atu^{-1}$	1
ktrUFP	rate of translation for folding-defective proteins	atu^{-1}	1
ktrXS	rate of XBP1 translation	atu^{-1}	1
ktrBiP	rate of BiP translation	atu^{-1}	1
ktrATF6	rate of ATF6 translation	atu^{-1}	1
ktrWFS	rate of WFS1 translation	atu^{-1}	1
ktrATF4	maximum rate of ATF4 mRNA transcription	atu^{-1}	1
ktrCHOP	rate of CHOP translation	atu^{-1}	1
ktrGADD34	rate of GADD34 translation	atu^{-1}	1
kdmXU	rate of degradation of unspliced XBP1 mRNA	atu^{-1}	1
kdmXS	rate of spliced XBP1 mRNA degradation	atu^{-1}	1
kdmBiP	rate of degradation of BiP mRNA	atu^{-1}	1
kdmWFS	rate of WFS1 mRNA degradation	atu^{-1}	1
kdmCHOP	rate of CHOP mRNA degradation	atu^{-1}	1
kdmGADD34	rate of GADD34 mRNA degradation	atu^{-1}	1
kdUFP	rate of degradation of unfolded proteins	atu^{-1}	0.1
kdXS	rate of XBP1 degradation	atu^{-1}	0.1
kdBiP	rate of BiP degradation	atu^{-1}	0.01
kdATF6	rate of ATF6 degradation	atu^{-1}	0.1
kdATF6GB	rate of degradation of ATF6 on Golgi membrane	atu^{-1}	0.1
kdATF6p50	rate of degradation of cytoplasmic domain of ATF6	atu^{-1}	0.1
kdWFS	rate of WFS1 degradation	atu^{-1}	0.1
kdATF4	rate of degradation of ATF4 protein	atu^{-1}	0.1
kdCHOP	rate of CHOP degradation	atu^{-1}	0.1
kdGADD34	rate of GADD34 degradation	atu^{-1}	0.1
ktrans	rate of transport of free ATF6 from ER to Golgi membrane	atu^{-1}	1
kcleave	rate of proteolytic cleavage of ATF6 by SP1 and 2 on Golgi membrane	atu^{-1}	10
kphos	maximum rate of phosphorylation of transport of eIF2α	atu^{-1}	5
kdephos	maximum rate of dephosphorylation of transport of eIF2α	atu^{-1}	0.5

Table S1: Species and parameters in the UPR model given in terms of arbitrary time units (*atu*) and arbitrary concentration units (*acu*).

Abbreviation	Description	Unit	Value / Initial
kdeAW	rate of ATF6 degradation regulated by WFS1	$acu^{-1}\, atu^{-1}$	1
kbu	rate of folding assisted by BiP	$acu^{-1}\, atu^{-1}$	0
kATF4	threshold for effective transcription by eIF2α	acu	0.1
J	binding affinity of activated PERK to eIF2α	acu	0.001
K	binding affinity of GADD34 and CReP to eIF2α	acu	0.001

Table S2: Reaction channels in the UPR model.

Group	Reaction	Rate Equation	Notes
1	$\emptyset \longrightarrow$ [UFPT]	ktrUFP [mUFPT]	
	[UFPT] $\longrightarrow \emptyset$	kdUFP [UFPT]	
	[UFPT] $\longrightarrow \emptyset$	kbu [BiP · UFP] [UFPT]	
2[†]	$\emptyset \longrightarrow$ [BiP · UFP]	kf [BiP] [UFP]	
	[UFPT] $\longrightarrow \emptyset$	kr [BiP · UFP]	
	$\emptyset \longrightarrow$ [BiP · IRE1α]	kf [BiP] [IRE1α]	
	$BiRE \longrightarrow \emptyset$	kr [BiP · IRE1α]	
	$\emptyset \longrightarrow$ [BiP · PERK]	kf [BiP] [PERK]	
	[BiP · PERK] $\longrightarrow \emptyset$	kr [BiP · PERK]	
	$\emptyset \longrightarrow$ [BiP · ATF6]	kf [BiP] [ATF6]	
	[BiP · ATF6] $\longrightarrow \emptyset$	kr [BiP · ATF6]	
	$\emptyset \longrightarrow$ [IRE1αA]	kf [IRE1α]^n	
	[IRE1αA] $\longrightarrow \emptyset$	kr [IRE1αA]	
	$\emptyset \longrightarrow$ [PERKA]	kf [PERK]^n	
	[PERKA] $\longrightarrow \emptyset$	kr [PERKA]	
3	$\emptyset \longrightarrow$ [mXbp1u]	$\text{trcXU}\,\frac{\text{basalXBP+kmAtfsXBP [ATF6p50]}}{\text{krcXU+basalXBP+kmAtfsXBP [ATF6p50]}}$	see Eqn. 13
	[mXbp1u] $\longrightarrow \emptyset$	kdmXU [mXbp1u]	
	[mXbp1u] $\longrightarrow$ [mXbp1s]	f_M([mXbp1u], 0.5 n [IRE1αA], krcSplice, ksplice)	$f_M(S_t, E_t, K_m, k_c)$ is the extended Michaelis-Menten equation given in Eqn. 10
	[mXbp1s] $\longrightarrow \emptyset$	kdmXS [mXbp1s]	
	$\emptyset \longrightarrow$ [Xbp1s]	ktrXS [mXbp1s]	
	[Xbp1s] $\longrightarrow \emptyset$	kdXS [Xbp1s]	
	$\emptyset \longrightarrow$ [mBiPT]	$\text{trcBiP} \times \frac{\text{basalBiP+kmXbp [Xbp1s]+kmAtfsBiP [ATF6p50]}}{\text{krcBiP+basalBiP+kmXbp [Xbp1s]+kmAtfsBiP [ATF6p50]}}$	see Eqn. 13
	[mBiPT] $\longrightarrow \emptyset$	kdmBiP [mBiPT]	
	$\emptyset \longrightarrow$ [BiPT]	ktrBiP[mBiPT]	
	[BiPT] $\longrightarrow \emptyset$	kdBiP [BiPT]	
4	$\emptyset \longrightarrow$ [ATF6T]	ktrATF6 [mATF6T]	
	[ATF6T] $\longrightarrow \emptyset$	kdATF6 [ATF6T]	
	[ATF6T] $\longrightarrow \emptyset$	kdeAW [WFS1] [ATF6T]	
	[ATF6T] $\longrightarrow$ [ATF6GB]	ktrans [ATF6]	
	[ATF6GB] $\longrightarrow \emptyset$	kdATF6GB [ATF6GB]	
	[ATF6GB] $\longrightarrow$ [ATF6p50]	kcleave [ATF6GB]	
	[ATF6p50] $\longrightarrow \emptyset$	kdATF6p50 [ATF6p50]	
	$\emptyset \longrightarrow$ [mWFS1]	$\text{trcWFS}\,\frac{\text{[ATF6p50]}}{\text{krcWFS+[ATF6p50]}}$	

Table S2: Reaction channels in the UPR model.

Group	Reaction	Rate Equation	Notes
	[mWFS1] $\longrightarrow \emptyset$	kdmWFS [mWFS1]	
	$\emptyset \longrightarrow$ [WFS1]	ktrWFS [mWFS1]	
	[WFS1] $\longrightarrow \emptyset$	kdWFS [WFS1]	
5‡	$\emptyset \longrightarrow$ [ATF4]	ktrATF4 [mATF4] $\frac{1}{1+([\mathrm{eIF2a}]/\mathrm{kATF4})^{nh}}$	
	[ATF4] $\longrightarrow \emptyset$	kdATF4 [ATF4]	
	$\emptyset \longrightarrow$ [mCHOP]	trcCHOP $\times$ $\frac{\mathrm{kmAtff}\,[\mathrm{ATF4}]+\mathrm{kmAtfs}\,[\mathrm{ATF6p50}]}{\mathrm{krcCHOP}+\mathrm{kmAtff}\,[\mathrm{ATF4}]+\mathrm{kmAtfs}\,[\mathrm{ATF6p50}]}$	see Eqn. 13
	[mCHOP] $\longrightarrow \emptyset$	kdmCHOP [mCHOP]	
	$\emptyset \longrightarrow$ [CHOP]	ktrCHOP [mCHOP]	
	[CHOP] $\longrightarrow \emptyset$	kdCHOP [CHOP]	
	$\emptyset \longrightarrow$ [mGADD34]	trcGADD34 $\frac{\mathrm{kmCHOP}\,[\mathrm{CHOP}]}{\mathrm{krcGADD34}+\mathrm{kmCHOP}\,[\mathrm{CHOP}]}$	
	[mGADD34] $\longrightarrow \emptyset$	kdmGADD34 [mGADD34]	
	$\emptyset \longrightarrow$ [GADD34]	ktrGADD34 [mGADD34]	
	[GADD34] $\longrightarrow \emptyset$	kdGADD34 [GADD34]	

† $[\mathrm{UFP}] = [\mathrm{UFPT}] - [\mathrm{BiP} \cdot \mathrm{UFP}]$
$[\mathrm{BiP}] = [\mathrm{BiPT}] - [\mathrm{BiP} \cdot \mathrm{UFP}] - [\mathrm{BiP} \cdot \mathrm{IRE1}\alpha] - [\mathrm{BiP} \cdot \mathrm{PERK}] - [\mathrm{BiP} \cdot \mathrm{ATF6}]$
$[\mathrm{IRE1}\alpha] = [\mathrm{IRE1}\alpha\mathrm{T}] - [\mathrm{BiP} \cdot \mathrm{IRE1}\alpha] - \mathrm{n}\,[\mathrm{IRE1}\alpha\mathrm{A}]$
$[\mathrm{PERK}] = [\mathrm{PERKT}] - [\mathrm{BiP} \cdot \mathrm{PERK}] - \mathrm{n}\,[\mathrm{PERKA}]$
$[\mathrm{ATF6}] = [\mathrm{ATF6T}] - [\mathrm{BiP} \cdot \mathrm{ATF6}]$

‡ $[\mathrm{eIF2a}] = [\mathrm{eIF2aT}] \times f_G(0.5\,\mathrm{kphos\,n}\,[\mathrm{PERKA}], \mathrm{kdephos}\,([\mathrm{GADD34}]+[\mathrm{CReP}]), \mathrm{J}/[\mathrm{eIF2aT}], \mathrm{K}/[\mathrm{eIF2aT}])$
$f_G(v,u,J,K) = \frac{2uK}{f_K(v,u,,J,K)+\sqrt{f_K(v,u,J,K)^2-4(v-u)uK}}$
$f_K(v,u,J,K) = v - u + vJ + uK$

1.1 Receptor Activation Model

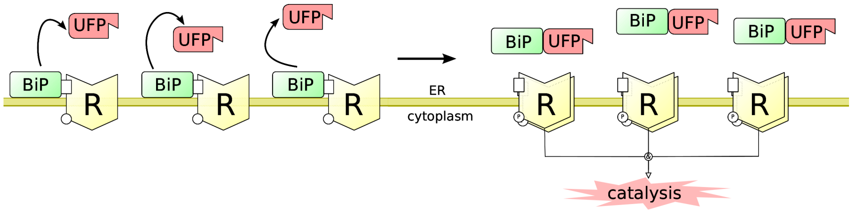

Group 2 of the set of reactions given in Table S2 corresponds to the activation dynamics of IRE1α, PERK and ATF6. We use the following ODE system (in XPP-AUT format) to investigate the parameter space of the module (IRE1α is given as an example):

```
# Differential equations:
dBiUFP/dt    = kf*BiP*UFP - kr*BiUFP
dBiRE1/dt    = kf*BiP*IRE1 - kr*BiRE1
dIRE1A/dt    = kf*ufpreg*IRE1^n - kr*IRE1A/(1.0+extIRE*UFP)

# Stoichiometric constraints:
UFP = UFPT - BiUFP
BiP = BiPT - BiRE1 - BiUFP
IRE1 = IRE1T - BiRE1 - n*IRE1A

# Logical switch for testing the effect of UFP on IRE1 activation:
ufpreg = IF(switch==1)then(UFP)else(1.0)
```

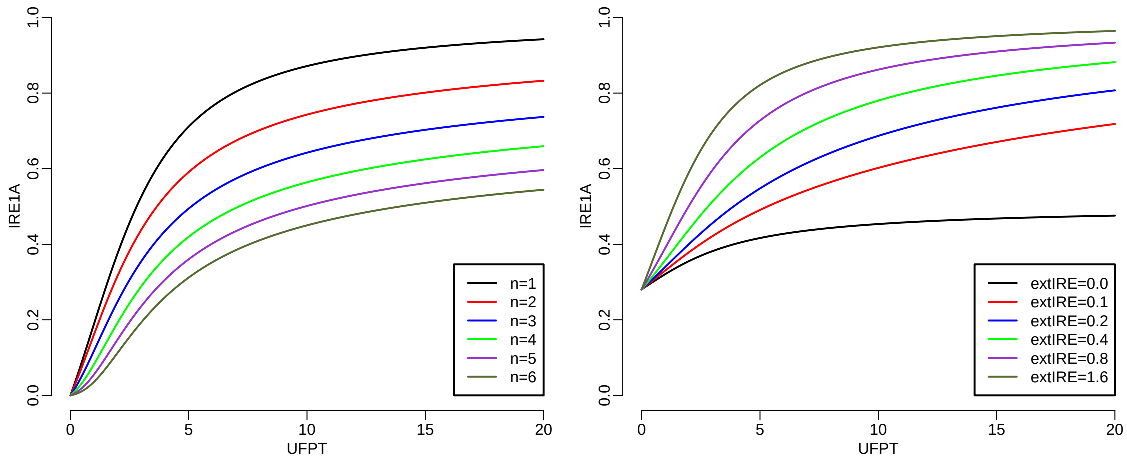

S1(a): UFP facilitates activation (switch = 1)

S1(b): UFP provides stability

Figure S1: Activation of the receptor complex (group 2 in Table S2) when UFP is directly involved. The plot in (c) displays the change in the amount of phosphorylated IRE1α with respect to UFP facilitated receptor activation, switch = 1. The plot in (d) displays the activation for different strengths, extIRE, of UFP stabilisation on the activated receptor complex. The reference model is given in section 1.1.

```
# Default parameters of the model:
par UFPT=0, BiPT=2, IRE1T=1
par kf=1, kr=1, n=1
par extIRE=0, switch=0

# Initial conditions:
init BiUFP=0, BiRE1=0, IRE1A=0

# Parameters for XPPAUT:
@ METH=STIFF, DSMAX=0.02, TOTAL=100
@ AUTOXMIN=0, AUTOXMAX=20, AUTOYMIN=0, AUTOYMAX=1
@ XLO=0, XHI=100, YLO=0, YHI=1
@ NPLOT=1, YP=IRE1A
done
```

1.2 IRE1α Branch

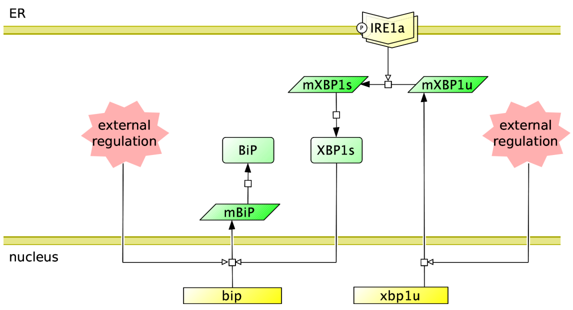

We combine group 3 of the reactions with group 2 (Table S2), in order to analyse the synthesis of chaperones in response to accumulation of UFP and the activation of the UPR.

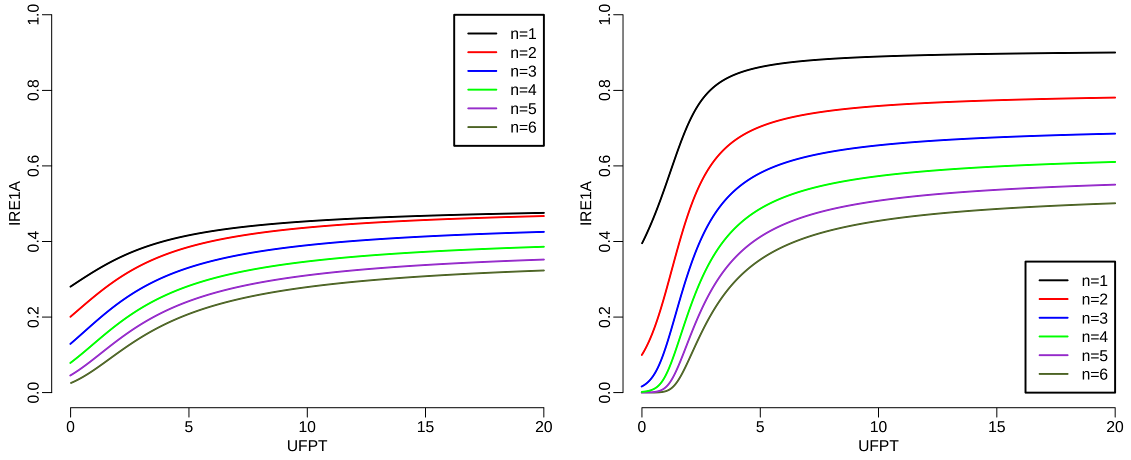

S2(a): reference

S2(b): stronger association (kf = 10)

Figure S2: Analysis of the receptor activation module without UFP involvement. The plot in (a) displays the change in the amount of phosphorylated IRE1α with respect to total UFP concentration. The plot in (b) presents the same analysis for stronger monomer binding, kf = 10. The reference model is given in section 1.1.

We investigate the dynamics of this module with the ODE system given below in XPP-AUT format.

```
# Differential equations:
dBiUFP/dt    = tmr*(kf*BiP*UFP - kr*BiUFP)
dBiRE1/dt    = tmr*(kf*BiP*IRE1 - kr*BiRE1)
dIRE1A/dt    = tmr*(kf*ufpreg*IRE1^n - kr*IRE1A/(1.0+extIRE*UFP))

dmXbp1u/dt   = trcXU*(basalXBP+extXBP)/(krcXU+basalXBP+extXBP) - kdmXU*
   mXbp1u - spliceRate
dmXbp1s/dt   = spliceRate - kdmXS*mXbp1s
dXbp1s/dt    = ktrXS*mXbp1s - kdXS*Xbp1s
dmBiPT/dt    = trcBiP*(basalBiP+kmXbp*Xbp1s+extBiP)/(krcBiP+basalBiP+kmXbp*
   Xbp1s+extBiP) - kdmBiP*mBiPT
dBiPT/dt     = ktrBiP*mBiPT - kdBiP*BiPT

ufpreg = IF(switch==1)then(UFP)else(1.0)

EMM(St,Et,Km,kcat) = 0.5*kcat*(St + Et + Km - sqrt((St + Et + Km)^2 - 4.0*
   St*Et))
spliceRate   = EMM(mXbp1u,0.5*n*IRE1A,krcSplice,ksplice)

# Stoichiometric constraints:
UFP = UFPT - BiUFP
BiP = BiPT - BiRE1 - BiUFP
IRE1 = IRE1T - BiRE1 - n*IRE1A

# Default parameters of the model:
par tmr=10
par UFPT=0, IRE1T=1
par kf=10, kr=1, n=4
par extIRE=0, switch=0
```

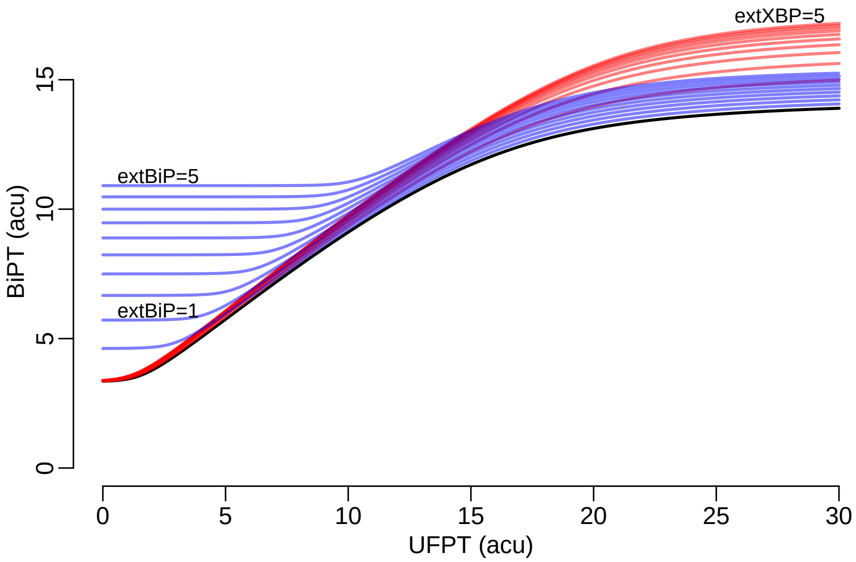

Figure S3: The level of total BiP with increasing UFP predicted by the IRE1α branch. The black solid line indicates the increase in the level of BiP when there is no external regulation of BiP or XBP1 mRNA. The red line indicates the deviation from this basal BiP regulation when the XBP1 mRNA expression is externally regulated with extXBP. The blue line indicates the change in BiP when the BiP mRNA is externally regulated with extBiP. The reference model is given in section 1.2.

```
par basalXBP=1, basalBiP=1
par extXBP=0, extBiP=0
par krcXU=5, krcBiP=5
par kmXbp=10
par ksplice=10, krcSplice=1
par trcXU=1, trcBiP=1
par ktrXS=1, ktrBiP=1
par kdmXU=1, kdmXS=1, kdmBiP=1
par kdXS=0.1, kdBiP=0.05

# Initial conditions:
init BiUFP=0, BiRE1=0, IRE1A=0
init mXbp1u=0, mXbp1s=0, Xbp1s=0, mBiPT=0, BiPT=0

# Parameters for XPPAUT:
@ METH=STIFF, BOUND=1000, TOTAL=100
@ AUTOXMIN=0, AUTOXMAX=2, AUTOYMIN=0, AUTOYMAX=3, AUTOVAR=kdmBiP
@ DS=0.02, DSMIN=0.001, DSMAX=0.02, PARMIN=0, PARMAX=30, NMAX=5000, NPR=50
@ XLO=0, XHI=100, YLO=0, YHI=3
@ NPLOT=5, YP=IRE1A, YP2=BiPT, YP3=Xbp1s, YP4=mXbp1u, YP5=mXbp1s
done
```

1.3 ATF6 Branch

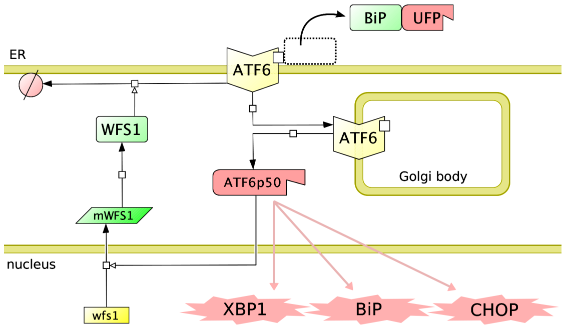

Here we add to the IRE1α branch another adaptive response pathway, the ATF6 branch. That is, we combine the groups 2, 3 and 4 in Table S2. We construct the ODE system of these interactions in XPP-AUT format as follows:

```
# Differential equations:
dBiUFP/dt    = tmr*(kf*BiP*UFP - kr*BiUFP)
dBiRE1/dt    = tmr*(kf*BiP*IRE1 - kr*BiRE1)
dBiATF/dt    = tmr*(kf*BiP*ATF6 - kr*BiATF)
dIRE1A/dt    = tmr*(kf*ufpreg*IRE1^n - kr*IRE1A/(1.0+extIRE*UFP))

dmXbp1u/dt   = trcXU*(basalXBP+kmAtfsXBP*ATF6p50)/(krcXU+basalXBP+kmAtfsXBP
    *ATF6p50) - kdmXU*mXbp1u - spliceRate
dmXbp1s/dt   = spliceRate - kdmXS*mXbp1s
dXbp1s/dt    = ktrXS*mXbp1s - kdXS*Xbp1s
dmBiPT/dt    = trcBiP*(basalBiP+kmXbp*Xbp1s+kmAtfsBiP*ATF6p50)/(krcBiP+
    basalBiP+kmXbp*Xbp1s+kmAtfsBiP*ATF6p50) - kdmBiP*mBiPT
dBiPT/dt     = ktrBiP*mBiPT - kdBiP*BiPT

dATF6T/dt    = ktrATF6*mATF6T - kdATF6*ATF6T - ktrans*ATF6 - kdeAW*WFS1*
    ATF6T
dATF6GB/dt   = ktrans*ATF6 - kdATF6GB*ATF6GB - kcleave*ATF6GB
dATF6p50/dt  = kcleave*ATF6GB - kdATF6p50*ATF6p50
dmWFS1/dt    = trcWFS*ATF6p50/(krcWFS+ATF6p50) - kdmWFS*mWFS1
dWFS1/dt     = ktrWFS*mWFS1 - kdWFS*WFS1

ufpreg = IF(switch==1)then(UFP)else(1.0)

EMM(St,Et,Km,kcat) = 0.5*kcat*(St + Et + Km - sqrt((St + Et + Km)^2 - 4.0*
    St*Et))
spliceRate   = EMM(mXbp1u,0.5*n*IRE1A,krcSplice,ksplice)

# Stoichiometric constraints:
UFP = UFPT - BiUFP
BiP = BiPT - BiRE1 - BiATF - BiUFP
IRE1 = IRE1T - BiRE1 - n*IRE1A
ATF6 = ATF6T - BiATF

# Default parameters of the model:
par tmr=10
par UFPT=0, IRE1T=1
par kf=10, kr=1, n=4
par extIRE=0, switch=0
par basalXBP=1, basalBiP=1
```

```
par krcXU=5, krcBiP=5, krcWFS=1
par kmXbp=10, kmAtfsXBP=10, kmAtfsBiP=1
par ksplice=10, krcSplice=1
par trcXU=1, trcBiP=1, trcWFS=1
par ktrXS=1, ktrBiP=1, ktrATF6=1, ktrWFS=1
par kdmXU=1, kdmXS=1, kdmBiP=1, kdmWFS=1
par kdXS=0.1, kdBiP=0.05, kdATF6=0.1, kdATF6GB=0.1, kdATF6p50=0.1, kdWFS
   =0.1
par mATF6T=5
par ktrans=1, kcleave=10
par kdeAW=1

# Initial conditions:
init BiUFP=0, BiRE1=0, BiATF=0, IRE1A=0
init mXbp1u=0, mXbp1s=0, Xbp1s=0, mBiPT=0, BiPT=0
init ATF6T=0, ATF6GB=0, ATF6p50=0, mWFS1=0, WFS1=0

# Parameters for XPPAUT:
@ METH=STIFF, BOUND=1000, TOTAL=100
@ AUTOXMIN=0, AUTOXMAX=2, AUTOYMIN=0, AUTOYMAX=3
@ DS=0.02, DSMIN=0.001, DSMAX=0.02, PARMIN=0, PARMAX=20, NMAX=1500, NPR=50
@ XLO=0, XHI=100, YLO=0, YHI=10
@ NPLOT=5, YP1=IRE1A, YP2=XBP1s, YP3=ATF6T, YP4=ATF6p50, YP5=BiPT
done
```

1.4 PERK Branch

1.4.1 The detailed model with genetic regulation

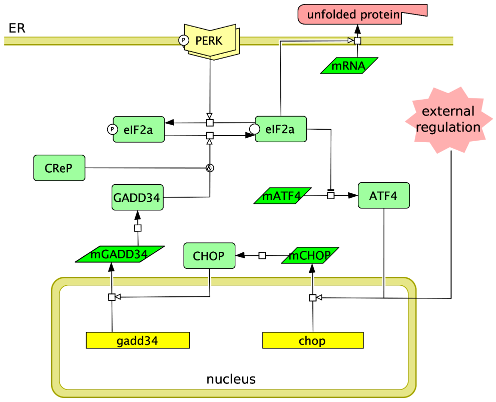

The set of reactions designated as group 5 in Table S2 is responsible for controlling transcription attenuation and apoptosis, for which PERK is the receptor molecule initiating signal transduction. We use the following ODE system (in XPP-AUT format) to investigate its parameter space:

```
# Differential equations:
dATF4/dt    = ktrATF4*mATF4/(1.0+(eIF2a/kATF4)^nh) - kdATF4*ATF4
dmCHOP/dt   = trcCHOP*(kmAtff*ATF4+extCHOP)/(krcCHOP+kmAtff*ATF4+extCHOP)
   - kdmCHOP*mCHOP
dCHOP/dt    = ktrCHOP*mCHOP - kdCHOP*CHOP
dmGADD34/dt = trcGADD34*(kmChop*CHOP)/(krcGADD34+kmChop*CHOP) - kdmGADD34*
   mGADD34
```

```
dGADD34/dt  = ktrGADD34*mGADD34 - kdGADD34*GADD34

Gamma(v,u,J,K) = v - u + v*J + u*K
fG(v,u,J,K) = 2*u*K/(Gamma(v,u,J,K) + sqrt(Gamma(v,u,J,K)^2 - 4*(v-u)*u*K)
    )
eIF2a = eIF2aT*fG(kphos*(PERKA),kdephos*(GADD34+CReP),J/eIF2aT,K/eIF2aT)

# Default parameters of the model:
par PERKA=0
par CReP=0
par mATF4=1
par eIF2aT=1
par nh=2
par J=0.001, K=0.001
par ktrATF4=1, ktrCHOP=1, ktrGADD34=1
par kdATF4=0.1, kdCHOP=0.1, kdGADD34=0.1
par trcCHOP=1, trcGADD34=1
par krcCHOP=1, krcGADD34=1
par kdmCHOP=1, kdmGADD34=1
par kphos=1, kdephos=1
par kATF4=0.1
par kmAtff=1, kmChop=1
par extCHOP=0

# Initial conditions:
init ATF4=0, mCHOP=0, CHOP=0, mGADD34=0, GADD34=0

# Parameters for XPPAUT:
aux eIF2a = eIF2a
@ BOUND=1000, TOTAL=200
@ DS=0.01, DSMIN=0.0001, DSMAX=0.1, NMAX=4000, NPR=400, PARMAX=20
@ AUTOXMIN=0, AUTOXMAX=20, AUTOYMIN=0, AUTOYMAX=10
@ XLO=0, XHI=200, YLO=0, YHI=10
@ NPLOT=3, YP1=ATF4, YP2=CHOP, YP3=GADD34
done
```

1.4.2 The reduced time-delay alternative

Much of the dynamics of the PERK branch can be summarised by a two-dimensional delay-differential equation (DDE):

$$\frac{d}{dt}[\text{ATF4}] = \frac{1}{1+[\text{eIF2a}]} - [\text{ATF4}]$$
$$\frac{d}{dt}[\text{GADD34}] = [\text{ATF4}]_{t-\tau} - [\text{GADD34}],$$

where

$$[\text{eIF2a}] = f_G\left(\text{PERKA}, [\text{GADD34}], 0.001, 0.001\right),$$

$f_G(v,u,j,k)$ is the Goldbeter-Koshland function [10], and PERKA is the concentration of the active catalytic subunit of PERK. This model encloses the genetic regulations between the translation of ATF4 and the activation of the negative regulator GADD34 in a time-delay, indicated by τ. The regulation of eIF2α is represented with zero-order ultrasensitivity, which is controlled by [GADD34] and the parameter PERKA.

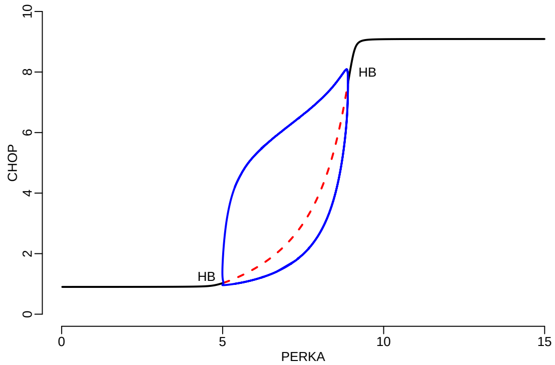

S4(a): oscillations in CHOP

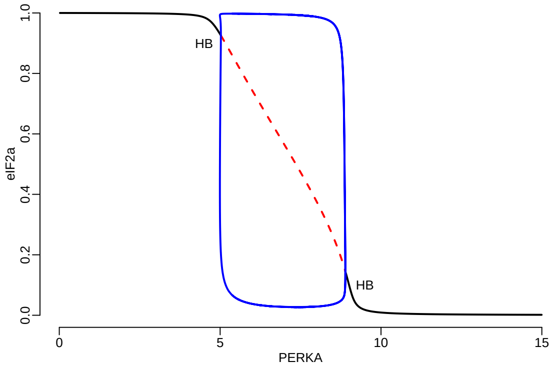

S4(b): oscillations in eIF2α

Figure S4: The bifurcation diagrams showing the persistence of oscillations in the detailed transcriptional regulation model. Solid black lines indicate stable stationary points for varying levels of PERKA. Dashed red lines indicate unstable stationary points, and the solid blue lines indicate the minimum and maximum values of CHOP (a), and eIF2α (b) oscillations. HB stands for Hopf bifurcation.

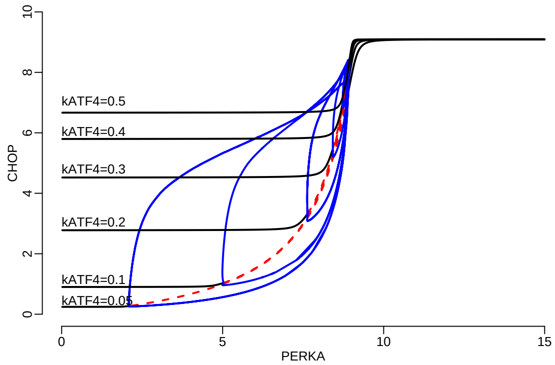

S5(a): kATF4

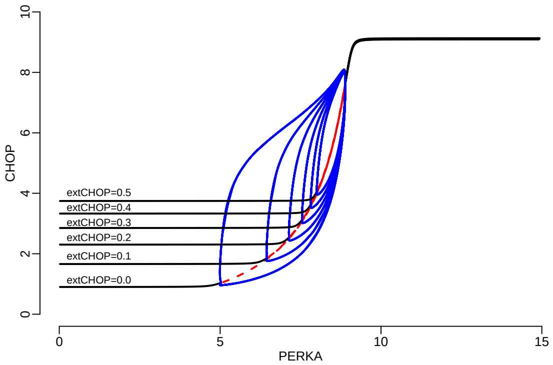

S5(b): extCHOP

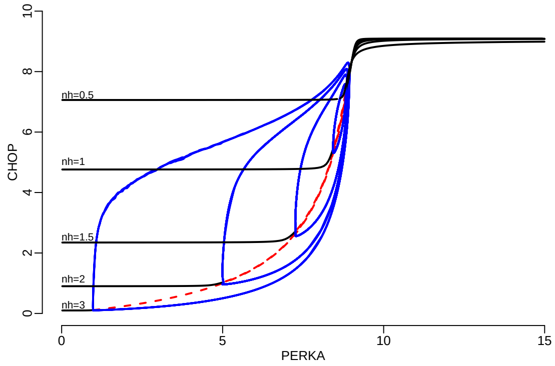

S5(c): nh

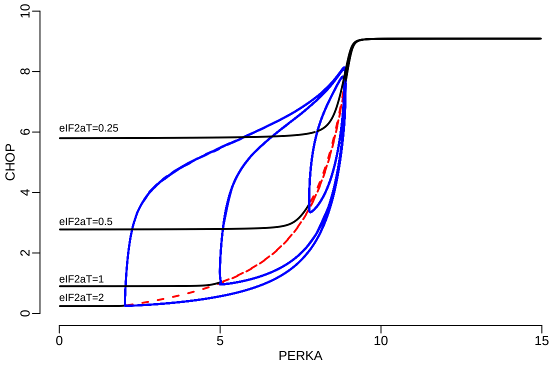

S5(d): eIF2αT

Figure S5: The bifurcation diagrams demonstrating the effects of kATF4 (a), extCHOP (b), nh (c) and eIF2aT (d) on the global behaviour of the PERK branch. The diagrams are drawn as in Figure S4.

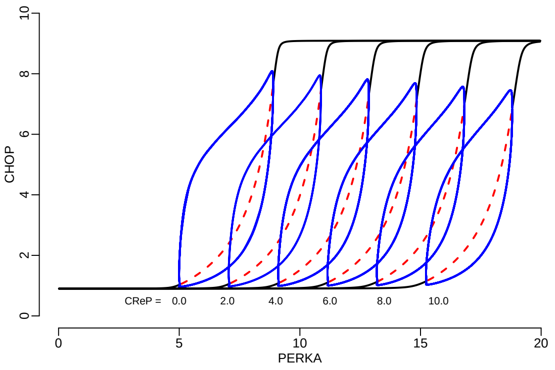

S6(a): CReP

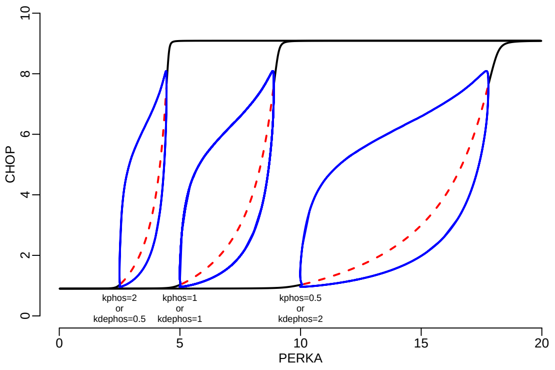

S6(b): kphos and kdephos

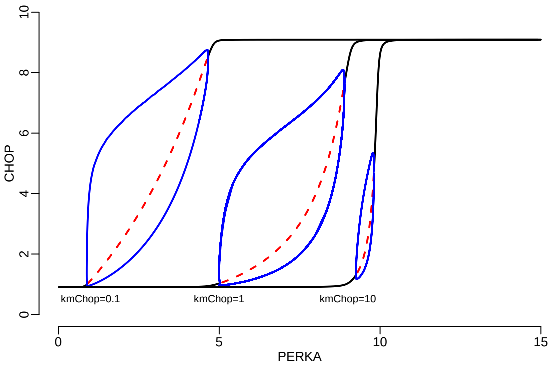

S6(c): kmChop

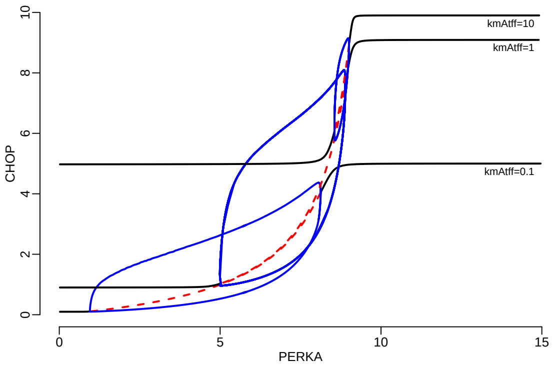

S6(d): kmAtff

Figure S6: The bifurcation diagrams demonstrating the effects of CReP (a), kphos and kdephos (b), kmChop (c) and kmAtff (d) on the global behaviour of the PERK branch. The diagrams are drawn as in Figure S4.

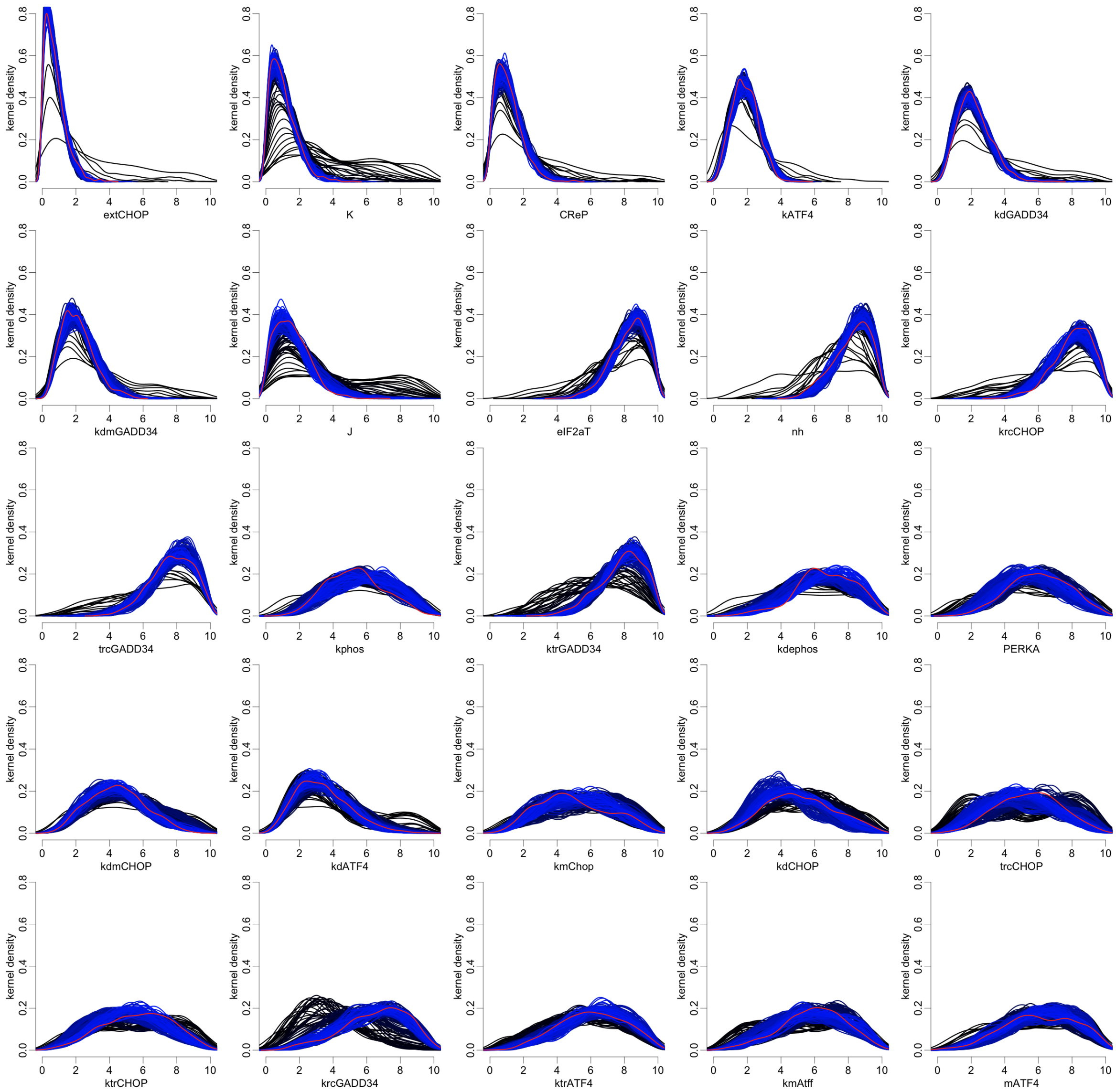

Figure S7: The marginal probability distributions of parameter values controlling the frequency of oscillations. Generations in the SMC algorithm are represented with a colour gradient from black to blue. The final generation is drawn in red. Kernel densities are estimated for each marginal distribution with a Gaussian smoothing kernel.

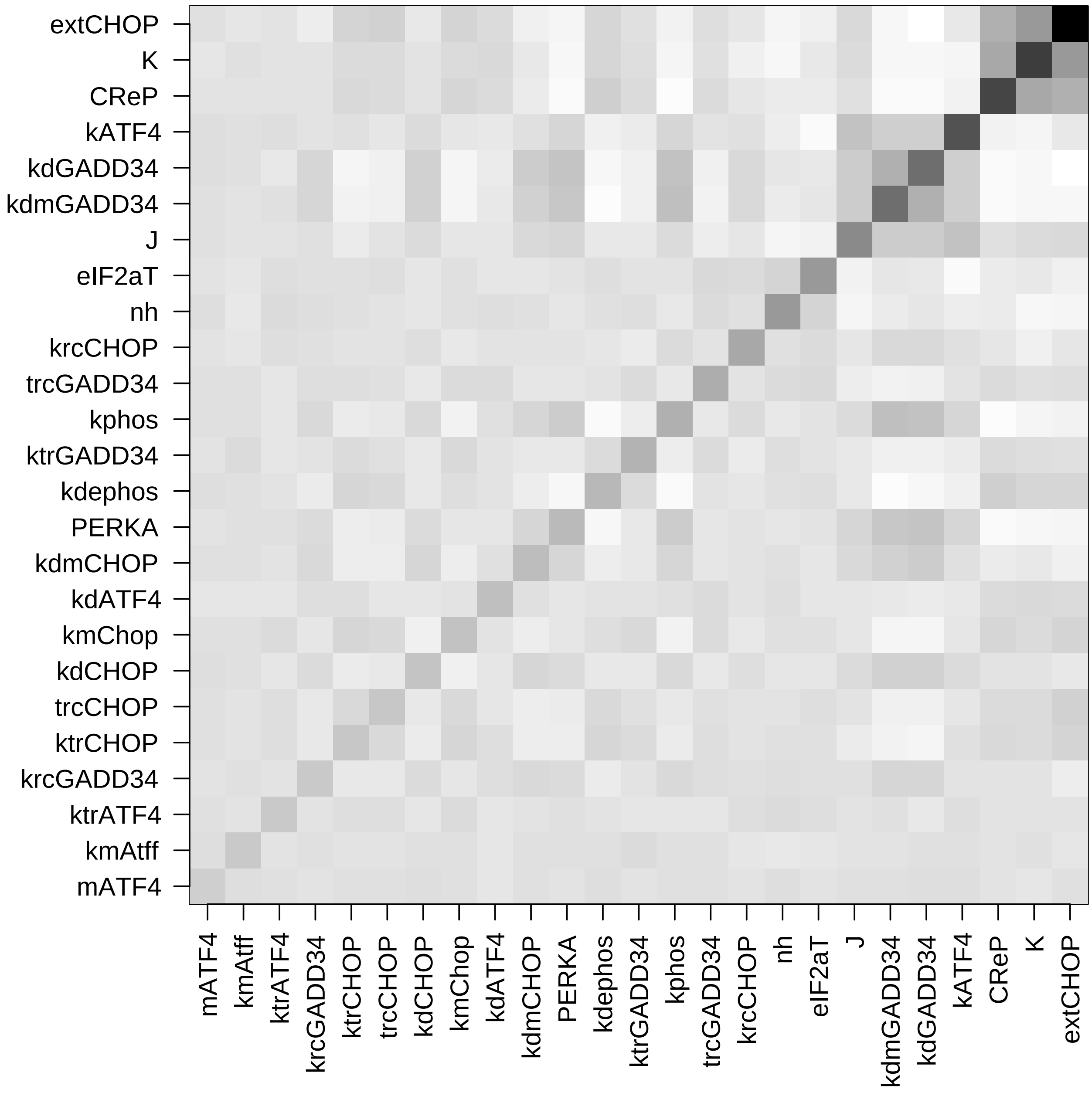

Figure S8: The sensitivity matrix obtained from the distribution of parameter values at the final generation of the SMC run. A population of 1000 individuals — with different sets of parameters — is evolved from possessing at least one peak in CHOP to an increasing number of minimally allowed peaks for 200 generations. Frequency of CHOP oscillations is calculated by dividing the average time between two peaks by the duration of the simulation. Each simulation is run until time 2000 with 0.1 unit intervals. The sensitivity matrix is calculated by inverting the covariance matrix of the probability distribution at the final generation [9]. Dark colours indicate higher relative sensitivities.

The model demonstrates an effective transformation from low activity to high activity states controlled by PERK (Fig. S9(a)). The transition when there is no time delay is linearly dependent on PERKA. When the time-delay as a result of genetic regulation is introduced, low and high activity states are preserved, but the intermediate state starts possessing oscillations. We observe that [ATF4] and [GADD34] levels oscillate between low and high activities within the intermediate range (Fig. S9(b)). We also observe that the amplitudes and the periods of these oscillations depend on the extent of the delay (Fig. S9(c)).

1.5 BAX Branch

The ODE model of the mitochondrial BAX/BAK/BH3 apoptosis pathway [11] can be given as follows:

$$\begin{aligned}
\frac{d}{dt}[\mathrm{Bcl2T}] &= \frac{\mathrm{kfbc}}{1+\mathrm{kmbc}\,[\mathrm{CHOP}]} - \mathrm{kdbc}\,[\mathrm{Bcl2T}] \\
\frac{d}{dt}[\mathrm{BAXmT}] &= (\mathrm{kfx} + \mathrm{kfx'}\,[\mathrm{BH3}])\,([\mathrm{BAXT}] - [\mathrm{BAXmT}]) - \mathrm{kbx}\,[\mathrm{BAXmT}] \\
\frac{d}{dt}[\mathrm{BH3T}] &= \mathrm{ks3} + \mathrm{kstr}\,\mathrm{ks3'}\,[\mathrm{CHOP}] - \mathrm{kd3}\,[\mathrm{BH3T}] \\
\frac{d}{dt}[\mathrm{BAXm{:}Bcl2}] &= \mathrm{kasx}\,[\mathrm{BAXm}]\,[\mathrm{Bcl2}] - (\mathrm{kdsx} + \mathrm{kbx})\,[\mathrm{BAXm{:}Bcl2}] \\
\frac{d}{dt}[\mathrm{BH3{:}Bcl2}] &= \mathrm{kas3}\,[\mathrm{BH3}]\,[\mathrm{Bcl2}] - (\mathrm{kds3} + \mathrm{kd3})\,[\mathrm{BH3{:}Bcl2}],
\end{aligned}$$

where

$$\begin{aligned}
[\mathrm{Bcl2}] &= [\mathrm{Bcl2T}] - [\mathrm{BH3{:}Bcl2}] - [\mathrm{BAXm{:}Bcl2}] \\
[\mathrm{BH3}] &= [\mathrm{BH3T}] - [\mathrm{BH3{:}Bcl2}] \\
[\mathrm{BAXm}] &= [\mathrm{BAXmT}] - [\mathrm{BAXm{:}Bcl2}].
\end{aligned}$$

1.6 The Importance of Oscillations

We have already shown that the oscillations seen at the intermediate state are generated by the difference in the time scales of eIF2α phosphorylation/dephosphorylation and the subsequent genetic regulation. Here, we further demonstrate the behaviour when the oscillations are prevented. To do this, we lower the binding affinities of the eIF2α kinase, PERK, and its phosphatases, GADD34 and CReP, and also adjust kstr to rearrange the intermediate activation states of CHOP and BAX. As a result, we observe in Figure S11 the intermediate activity state without translational activity in contrast to the case with oscillations (see the main text).

2 The Extended Michaelis-Menten Model

The canonical Michaelis-Menten equation describes the set of chemical reactions

$$E + S \underset{k_f}{\overset{k_d}{\leftrightarrows}} ES \xrightarrow{k_{cat}} E + P,$$

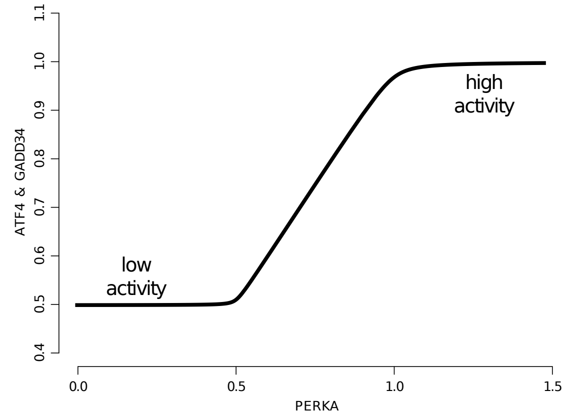

S9(a): no delay

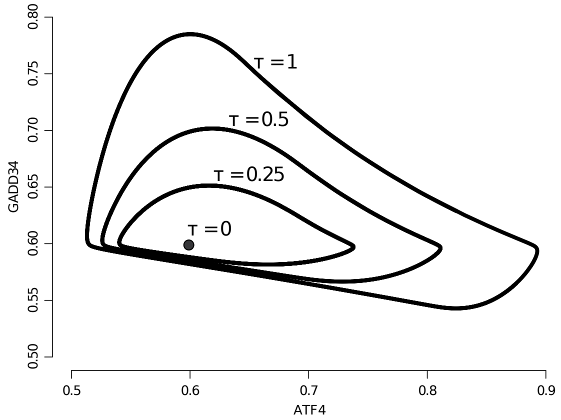

S9(b): delay

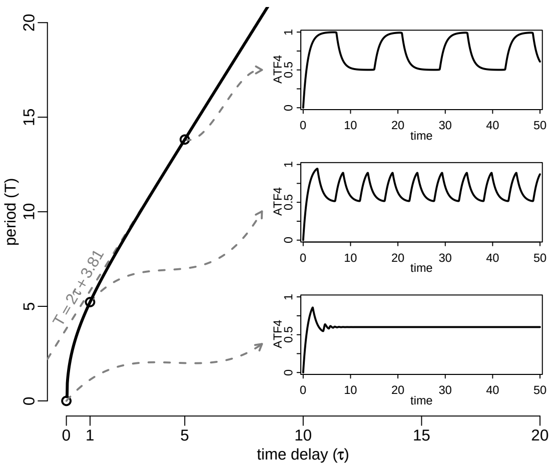

S9(c): period

Figure S9: Activation of the PERK branch and the control of translation attenuation. (a) Two states of activity is shown where transition from low to high ATF4 and GADD34 is managed by PERK. (b) The dependence of oscillations on the time-delay caused by transcription and translation. (c) The period of oscillations, T, is shown with respect to the extent of the time-delay, τ. Also in the inset, trajectories of ATF4 are shown for $\tau = \{0, 1, 5\}$. For (b) and (c), PERKA is fixed to 0.6.

Table S3: Rescaled parameters of the apoptosis model [12, 11].

**Name**	**Unit**	**Value**
BAXT	acu	100
kfbc	$acu\, atu^{-1}$	10
kdbc	atu^{-1}	0.1
kmbc	·	0.03
kstr	·	0.2
kfx	atu^{-1}	1
kfxp	$acu^{-1}\ atu^{-1}$	3
kbx	atu^{-1}	2
kasx	$acu^{-1}\ atu^{-1}$	90
kdsx	atu^{-1}	0.05
ks3	$acu\, atu^{-1}$	0.1
ks3p	atu^{-1}	0.6
kd3	atu^{-1}	0.01
kas3	$acu^{-1}\ atu^{-1}$	10
kds3	atu^{-1}	0.01

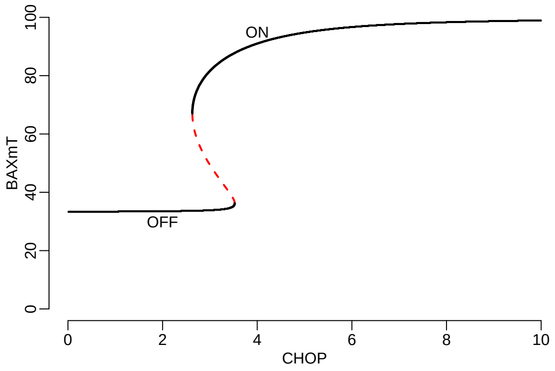

S10(a): bifurcation diagram

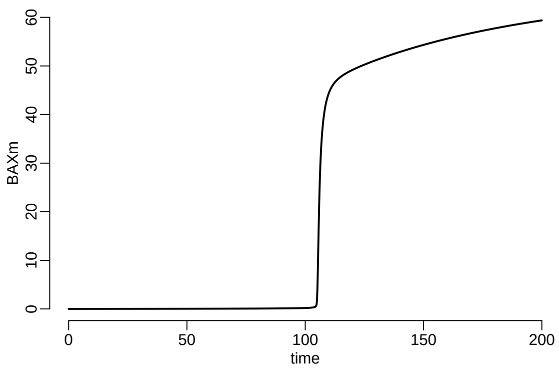

S10(b): temporal behaviour

Figure S10: Dependence of apoptosis on the level of CHOP. (a) The signal-response curve plotting the change in the total BAX levels on mitochondrial outer membrane at steady-state. The dashed red line shows the unstable fixed points which separate the “off” and “on” states. (b) The activation of apoptosis shown as the time-dependent rise in active BAX on mitochondrial outer membrane (CHOP=5).

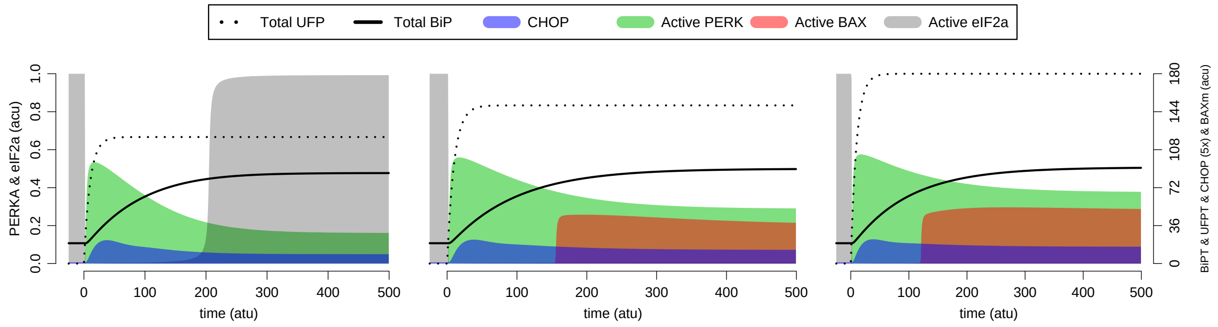

S11(a): mild stress S11(b): moderate stress S11(c): severe stress

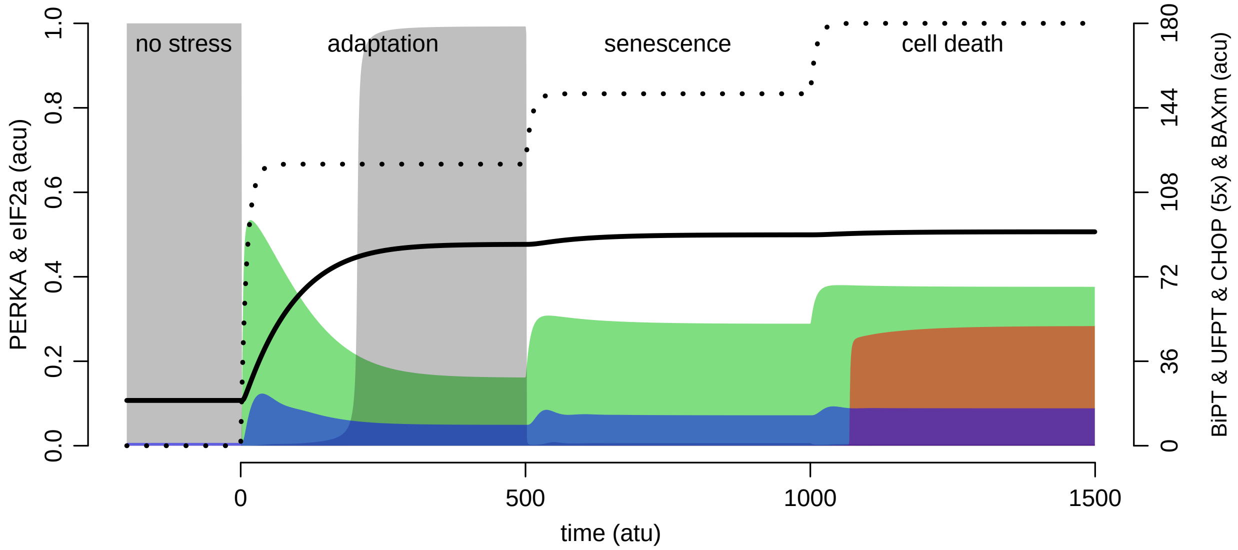

S11(d): progressive stimulation

Figure S11: The low, intermediate and high activity states without oscillations. The three states of activation are given in (a-c) as administered separately, and in (d) sequentially. The plots are drawn as in Figure 12 and 13 in the main text. The parameters are identical with the exception of the following three: $J = K = 0.05$, kstr $= 0.25$. The legend is shown on top of the plots.

where $[E]$ denotes the free enzyme, $[S]$ denotes the substrate, $[P]$ denotes the product, and $[ES]$ denotes the enzyme-bound substrate concentration at a given time. Using the rule of conservation of mass, the set of differential equations describing the full system can be written as

$$\frac{d}{dt}[E] = k_c[ES] + k_d[ES] - k_f[E][S] \tag{1}$$

$$\frac{d}{dt}[S] = k_d[ES] - k_f[E][S] \tag{2}$$

$$\frac{d}{dt}[ES] = k_f[E][S] - k_c[ES] - k_d[ES] \tag{3}$$

$$\frac{d}{dt}[P] = k_c[ES]. \tag{4}$$

If we assume fast reaction kinetics,

$$\frac{d}{dt}[ES] = 0 = k_f[E][S] - k_c[ES] - k_d[ES],$$

therefore,

$$[ES] = \frac{k_f}{k_c + k_d}[E][S] = \frac{1}{K_m}[E][S],$$

where the enzyme affinity parameter $K_m = (k_c + k_d)/k_f$.

We define E_t as the total enzyme concentration,

$$E_t = [E] + [ES] \tag{5}$$

$$[ES] = E_t - [E] = E_t - K_m[ES]\frac{1}{[S]} \tag{6}$$

$$[ES] = \frac{E_t[S]}{K_m + [S]}. \tag{7}$$

In addition, we define S_t as the total substrate concentration at any time point,

$$S_t = [S] + [ES] \tag{8}$$

$$S_t = \frac{E_t[S]}{K_m + [S]} + [S]. \tag{9}$$

Keeping in mind that the concentration values are positive real numbers, we can write

$$[S] = \frac{1}{2}\left(S_t - E_t - K_m + \sqrt{(S_t + E_t + K_m)^2 - 4\,S_t\,E_t}\right).$$

Consequently, we can convert the reaction system into

$$S \longrightarrow P,$$

where

$$\frac{d}{dt}[P] = \frac{1}{2}k_c\left(S_t + E_t + K_m - \sqrt{(S_t + E_t + K_m)^2 - 4\,S_t\,E_t}\right). \tag{10}$$

Unlike conventional Michaelis-Menten equation, this version handles high enzyme concentrations better. The dependency of the rate of catalysis on the concentration of the enzyme

is not linear with this equation. Also, it assumes the substrate concentration given will be distributed into free substrate and its enzyme bound form, and calculates them intrinsically. The conventional equation, however, assumes the substrate concentration given excludes the enzyme bound substrate, which would have already formed in the reaction medium. As expected, this equation approaches the conventional form as the enzyme concentration is lower than that of the substrate. Although it could be relatively more laborious to work with, we believe that its benefits outweigh its limitations, and therefore, use it in our UPR model.

3 Genetic Regulation Model

Here, we aim to derive a simple, general-purpose mathematical equation to be used when many effectors are expected to regulate a low-copy gene. Our model is rooted strongly to the molecular biology of transcription, and it is based on Hill-type kinetics [13, 14, 15]. To begin with, we investigate the following canonical reaction system,

$$\mathrm{DNA} + \mathrm{TF}_i \underset{k_{ri}}{\overset{k_{fi}}{\rightleftharpoons}} \mathrm{DNA} \cdot \mathrm{TF}_i \overset{k_{ci}}{\rightarrow} \mathrm{DNA} + \mathrm{TF}_i + \mathrm{mRNA},$$

where DNA represents the gene, more specifically the promoter region, whose transcription product is the mRNA in the final reaction. We assume that the underlying complexity for the transcription of a gene with a transcription factor (TF_i in the reaction for different transcription factors) can be simplified as above.

According to this reaction system, the probability of transcription, *i.e.* increasing the mRNA count by 1, in an infinitesimal time interval can be written as,

$$\frac{d}{dt}\,\mathrm{Pr}(\mathrm{mRNA}|\mathrm{mRNA}-1) = \sum_i k_{ci}\,\mathrm{Pr}(\mathrm{DNA}\cdot\mathrm{TF}_i). \tag{11}$$

For a specific transcription factor, TF_i,

$$\frac{d}{dt}\,\mathrm{Pr}(\mathrm{DNA}\cdot\mathrm{TF}_i) = k_{fi}\mathrm{TF}_i\,\mathrm{Pr}(\mathrm{DNA}) - (k_{ri}+k_{ci})\,\mathrm{Pr}(\mathrm{DNA}\cdot\mathrm{TF}_i).$$

We assume that there is a single copy of the gene and a single transcription initiation machinery, which could be occupied by one transcription factor at a time. Therefore, the transcription machinery is either unassembled leaving the DNA bare, or it is formed in combination with a transcription factor. This assumption implies that

$$\mathrm{Pr}(\mathrm{DNA}) + \sum_j \mathrm{Pr}(\mathrm{DNA}\cdot\mathrm{TF}_j) = 1 \tag{12}$$

at any time point.

Initiation is the rate-limiting step compared to the rest of the transcription process, and the transcription factor is released immediately after the initiation is complete. The promoter region is available for binding immediately after it is cleared off the previous initiation complex [16]. Based on this, we assume that the promoter region and the transcription factors attain a pseudo-steady-state independent of the rest of the transcription process, *i.e.* the process of transcription is adiabatically decoupled from the regulation of transcription.

Therefore, the probability of observing the TF bound to the DNA is virtually constant over time:

$$\frac{d}{dt}\,\mathrm{Pr}(\mathrm{DNA}\cdot\mathrm{TF}_i) = 0 \quad \text{for any } i \in \{1,2,3,\dots\}.$$

Equation (12) then becomes,

$$\mathrm{Pr}(\mathrm{DNA}) + \sum_j \frac{k_{fj}}{k_{rj}+k_{cj}}\mathrm{TF}_j\,\mathrm{Pr}(\mathrm{DNA}) = 1,$$

$$\mathrm{Pr}(\mathrm{DNA}) = \frac{1}{1+\sum_j \mathrm{TF}_j/K_{m_j}} \quad \text{and} \quad \mathrm{Pr}(\mathrm{DNA}\cdot\mathrm{TF}_i) = \frac{\mathrm{TF}_i/K_{m_i}}{1+\sum_j \mathrm{TF}_j/K_{m_j}},$$

where $K_{m_i} = (k_{ri}+k_{ci})/k_{fi}$, for the ith transcription factor, corresponds to the well-known "affinity" parameter used in Michaelis-Menten kinetics. Equation (11) then becomes,

$$\frac{d}{dt}\,\mathrm{Pr}(\mathrm{mRNA}|\mathrm{mRNA}-1) = \frac{\sum_i k_{ci}\,\mathrm{TF}_i/K_{m_i}}{1+\sum_j \mathrm{TF}_j/K_{m_j}}.$$

For mRNA$\gg 1$ and TF$\gg 1$ the rate of mRNA synthesis can be written as a deterministic ordinary differential equation [1]:

$$\frac{d}{dt}[\mathrm{mRNA}] = \frac{\sum_i k_{ci}\,[\mathrm{TF}]_i/K_{m_i}}{1+\sum_j [\mathrm{TF}]_j/K_{m_j}}, \tag{13}$$

where [] represents species concentration.

There are reports about burst-like transcription, which state that once the initiation is complete many mRNA molecules get transcribed [17, 18]. The assumptions and the model we derive do not contradict with such observations. If desired, model parameters can be adjusted to match this dynamics.

For the sake of restricting the complexity of the UPR model, we employ an additional assumption stating that

- for a specific gene, the rates of transcription, k_c, and the binding affinity, K_m, for each regulator (transcription factor / activator / inhibitor) are the same.

Therefore, the parameters with names beginning with "trc" or "krc" contribute to k_c or K_m, respectively. We make exceptions to this assumption for some of the regulators, such as the relative contributions of ATF4 and ATF6p50 to CHOP activation, which can be seen in Tables S1 and S2.

References

[1] Rao C, Arkin A: **Stochastic chemical kinetics and the quasi-steady-state assumption: Application to the Gillespie ...** . *The Journal of chemical physics* 2003, [http://sahara.lbl.gov/~taltman/Published/Rao_CV_Stochastic_chemical_kinetics_reprint.pdf].

[2] López-Lastra M, Rivas A, Barría MI: **Protein synthesis in eukaryotes: the growing biological relevance of cap-independent translation initiation**. *Biol Res* 2005, **38**(2-3):121–46.

[3] Ron D, Walter P: **Signal integration in the endoplasmic reticulum unfolded protein response**. *Nat Rev Mol Cell Biol* 2007, **8**(7):519–29, [http://www.nature.com/nrm/journal/v8/n7/full/nrm2199.html].

[4] Lee AH, Iwakoshi NN, Glimcher LH: **XBP-1 regulates a subset of endoplasmic reticulum resident chaperone genes in the unfolded protein response**. *Molecular and Cellular Biology* 2003, **23**(21):7448–59.

[5] Chakrabarti A, Chen AW, Varner JD: **A review of the mammalian unfolded protein response**. *Biotechnol. Bioeng.* 2011, **108**(12):2777–93.

[6] Yamamoto K, Yoshida H, Kokame K, Kaufman RJ, Mori K: **Differential contributions of ATF6 and XBP1 to the activation of endoplasmic reticulum stress-responsive cis-acting elements ERSE, UPRE and ERSE-II**. *Journal of Biochemistry* 2004, **136**(3):343–50.

[7] Yoshida H, Matsui T, Yamamoto A, Okada T, Mori K: **XBP1 mRNA is induced by ATF6 and spliced by IRE1 in response to ER stress to produce a highly active transcription factor**. *Cell* 2001, **107**(7):881–91.

[8] Harding HP, Novoa I, Zhang Y, Zeng H, Wek R, Schapira M, Ron D: **Regulated translation initiation controls stress-induced gene expression in mammalian cells**. *Molecular Cell* 2000, **6**(5):1099–108.

[9] Erguler K, Stumpf MPH: **Practical limits for reverse engineering of dynamical systems: a statistical analysis of sensitivity and parameter inferability in systems biology models**. *Mol. BioSyst.* 2011, **7**(5):1593–602.

[10] Goldbeter A, Koshland D: **An Amplified Sensitivity Arising from Covalent Modification in Biological Systems**. *Proceedings of the National Academy of Sciences of the ...* 1981, [http://links.jstor.org/sici?sici=0027-8424(198111)78%253A11%253C6840%253AAASAFC%253E2.0.CO%253B2-%2523].

[11] Tyson JJ, Baumann WT, Chen C, Verdugo A, Tavassoly I, Wang Y, Weiner LM, Clarke R: **Dynamic modelling of oestrogen signalling and cell fate in breast cancer cells**. *Nat Rev Cancer* 2011, **11**(7):523–32, [http://www.nature.com/nrc/journal/v11/n7/full/nrc3081.html].

[12] Zhang T, Brazhnik P, Tyson JJ: **Computational analysis of dynamical responses to the intrinsic pathway of programmed cell death**. *Biophysical Journal* 2009, **97**(2):415–34.

[13] Kaern M, Elston TC, Blake WJ, Collins JJ: **Stochasticity in gene expression: from theories to phenotypes**. *Nat Rev Genet* 2005, **6**(6):451–64.

[14] Cao J, Zhao H: **Estimating dynamic models for gene regulation networks**. *Bioinformatics* 2008, **24**(14):1619, [http://bioinformatics.oxfordjournals.org/cgi/content/abstract/24/14/1619].

[15] Ribeiro AS: **Stochastic and delayed stochastic models of gene expression and regulation**. *Mathematical biosciences* 2010, **223**:1–11.

[16] Shahrezaei V, Swain P: **The stochastic nature of biochemical networks**. *Current Opinion in Biotechnology* 2008, **19**(4):369–374, [http://www.sciencedirect.com/science?_ob=ArticleURL&_udi=B6VRV-4T5V6XF-1&_user=10&_coverDate=08/31/2008&_rdoc=13&_fmt=high&_orig=browse&_srch=doc-info(%2523toc%25236244%25232008%2523999809995%2523696340%2523FLA%2523display%2523Volume)&_cdi=6244&_sort=d&_docanchor=&_ct=18&_version=1&_urlVersion=0&_userid=10&md5=324c9e7276db30b63f014093da5acead].

[17] Golding I, Paulsson J, Zawilski SM, Cox EC: **Real-time kinetics of gene activity in individual bacteria**. *Cell* 2005, **123**(6):1025–36.

[18] Mitarai N, Dodd IB, Crooks MT, Sneppen K, Margalit H: **The Generation of Promoter-Mediated Transcriptional Noise in Bacteria**. *PLoS Computational Biology* 2008, **4**(7):e1000109, [http://www.ploscompbiol.org/article/info:doi/10.1371/journal.pcbi.1000109].
